# Utilisation of endocrine therapy for cancer in Indigenous peoples: a systematic review and meta-analysis

**DOI:** 10.1186/s12885-024-12627-6

**Published:** 2024-07-22

**Authors:** Habtamu Mellie Bizuayehu, Sewunet Admasu Belachew, Shafkat Jahan, Abbey Diaz, Siddhartha Baxi, Kalinda Griffiths, Gail Garvey

**Affiliations:** 1 First Nations Cancer and Wellbeing Research Program, School of Public Health, The University of Queensland, Brisbane, Australia; 2 GenesisCare Australia, Griffith University, Gold Coast, Australia; 3 Poche SA+NT, Flinders University, Darwin, Australia; 4 Menzies School of Health Research, Darwin, Australia; 5 Centre for Big Data Research in Health, UNSW, Sydney, Australia

**Keywords:** Cancer, Endocrine treatment, Ethnicity, First Nations peoples, Indigenous peoples, Systematic review

## Abstract

**Background:**

Indigenous peoples worldwide experience inequitable cancer outcomes, and it is unclear if this is underpinned by differences in or inadequate use of endocrine treatment (ET), often used in conjunction with other cancer treatments. Previous studies examining ET use in Indigenous peoples have predominately focused on the sub-national level, often resulting in small sample sizes with limited statistical power. This systematic review aimed to collate the findings ofarticles on ET utilisation for Indigenous cancer patients and describe relevant factors that may influence ET use.

**Methods:**

We conducted a systematic review and meta-analysis of studies reporting ET use for cancer among Indigenous populations worldwide. PubMed, Scopus, CINAHL, Web of Science, and Embase were searched for relevant articles. A random-effect meta-analysis was used to pool proportions of ET use. We also performed a subgroup analysis (such as with sample sizes) and a meta-regression to explore the potential sources of heterogeneity. A socio-ecological model was used to present relevant factors that could impact ET use.

**Results:**

Thirteen articles reported ET utilisation among Indigenous populations, yielding a pooled estimate of 67% (95% CI:54 − 80), which is comparable to that of Indigenous populations 67% (95% CI: 53 − 81). However, among studies with sufficiently sized study sample/cohorts (≥ 500), Indigenous populations had a 14% (62%; 95% CI:43 − 82) lower ET utilisation than non-Indigenous populations (76%; 95% CI: 60 − 92). The ET rate in Indigenous peoples of the USA (e.g., American Indian) and New Zealand (e.g., Māori) was 72% (95% CI:56–88) and 60% (95% CI:49–71), respectively. Compared to non-Indigenous populations, a higher proportion of Indigenous populations were diagnosed with advanced cancer, at younger age, had limited access to health services, lower socio-economic status, and a higher prevalence of comorbidities.

**Conclusions:**

Indigenous cancer patients have lower ET utilisation than non-Indigenous cancer patients, despite the higher rate of advanced cancer at diagnosis. While reasons for these disparities are unclear, they are likely reflecting, at least to some degree, inequitable access to cancer treatment services. Strengthening the provision of and access to culturally appropriate cancer care and treatment services may enhance ET utilisation in Indigenous population. This study protocol was registered on Prospero (CRD42023403562).

**Supplementary Information:**

The online version contains supplementary material available at 10.1186/s12885-024-12627-6.

## Background

Cancer is the leading cause of premature death globally in 2021, ranking first in 57 countries and second in 70 countries [1]. In 2020, it caused approximately 10 million deaths worldwide. In 2040, new cancer cases are expected to increase by 47% to 28.4 million compared to 19.3 million in 2020 [2].

Indigenous peoples (a term respectfully used here to refer to the original inhabitants of colonised/occupied societies who have retained social, cultural, economic and political characteristics that are distinctive from that of the dominant society [3]) are minority population groups. Compared to other populations, Indigenous peoples have a higher risk of cancer morbidity and mortality [4]. For instance, in 2011–2015, Indigenous Australians had a higher incidence of cancer (495 per 100,000) compared to other Australians (472 per 100,000), and in 2010–2019, cancer mortality increased by 12% among Indigenous peoples while decreasing by 10% in other Australians [5]. Similarly, the incidence rate among the Southwest American Indians and Alaska Natives American Indian/Alaskan Native population in 2022 was 49% higher than other Americans [6, 7]. In New Zealand, the cancer mortality rate between 1981 − 2011 did not significantly decrease in the Māori and Pacific Islander peoples but decreased significantly in other New Zealanders [8]. For instance, between 2006 and 2011, the incidence rate of cancer per 100,000 females was 202 for Māori and 134.8 for Pacific Islander peoples, which was higher compared to other New Zealanders (92.6). Similarly, the incidence rate of cancer per 100,000 males was 214.3 for Māori and 148.4 for Pacific Islander peoples, higher than the estimates for other New Zealanders (112.2).

Enhancing the utilisation of cancer treatment options, including endocrine treatment (ET), is one of the strategies for reducing the existing disparity in cancer outcomes [4–9]. ETs, including estrogen receptor modulators, aromatase inhibitors, and gonadotropin-releasing hormone agonists, are often prescribed as an adjunct to other cancer treatments to reduce cancer cell growth, metastasis, and recurrence, and generally have relatively lower side effects than other cancer treatment options, such as chemotherapy. ET also has other benefits, including preserving the fertility of women and reducing cost and burden on the health system (e.g. reduced medical visits to manage treatment-related side effects) [8, 10–15]. Furthermore, ETs are generally safe for extended use (five years and beyond). However, medication adherence for chronic conditions remains a major issue among Indigenous peoples, as demonstrated in a review of Indigenous Australians [16].

Despite ET’s importance in cancer care, its utilisation is affected by a multitude of factors at the level of the individual (such as age, weight, sex, race/ethnicity, cancer stage, grade, and other comorbidities, educational status, income, private health insurance, smoking, alcohol use, individual perception), interpersonal (such as social support, familiarity/interaction with health professionals, health professionals’ perception, cultural similarity) and community/organisational (such as distance to hospital, remoteness, health workers’ expertise/training, guidelines/protocols, patient support service availability and hospital type) [15, 17–29]. For instance, a systematic review focused on race/ethnic groups (including the Indigenous peoples) found a higher utilisation of ET among older aged groups compared to younger counterparts. Additionally, ET was more commonly usedamong individuals with advanced cancer types, private health insurance, positive perceptions about treatment importance, perceived efficacy in patient-provider interactions and when primarily provided by medical oncology specialist rather than surgeons. Conversely, ETutilisation was lower among those with other comorbidities and facing higher out-of-pocket costs [29].

While there are studies assessing ET utilisation among the Indigenous peoples, the majority have reported on subnational level data with small sample sizes ranging from 50 to 150 [26, 30–36]. For instance, the sample size of American Indian/Alaskan Native (*n* = 77) was much smaller compared to non-Indigenous populations (*n* = 16,677), according to a sub-national study in the USA [31]. However, this limitation can be partly addressed by conducting a meta-analysis to pool the estimates of ET use among Indigenous peoples reported in individual studies [37–39], an essential component of the current study. By including studies from diverse geographic regions and populations, a meta-analysis can enhance the precision and generalisability of the findings at a global level. In addition, identifying the various factors influencing the utilisation of ET at the individual, interpersonal and community/organisational-levels [15, 17–29] is crucial for providing the context to the observed practices. This altogether provides a broader perspective on ET use among Indigenous peoples worldwide, allowing for a more comprehensive understanding on the topic.

Ensuring equitable healthcare and addressing health disparities for Indigenous peoples, including ET utilisation, is a top priority on the global public health agenda [4, 40]. Therefore, the findings could be used as a crucial piece of information by policymakers and other stakeholders working with Indigenous peoples in their efforts to implement existing and/or enact new policies to redress unwarranted disparities in treatment use. Underrepresentation of the Indigenous people in research and evidence-based practice is one of the ongoing challenges [4, 40–43], and this review will inform the global evidence and strengthen researchers’ efforts to conduct culturally acceptable and person-tailored effective interventions to improve ET utilisation, ultimately ensuring equity of cancer outcomes.

## Methods

### Protocol registration

The review followed the Preferred Reporting Items for Systematic Reviews and Meta-Analysis (PRISMA) guidelines (see Additional file 1) [44], and the study protocol was registered on Prospero (CRD42023403562).

### Review question

The PECO (Population, Exposure, Comparator and Outcome) framework was used to structure the review question [45]. Individuals diagnosed with cancer (Population) reporting endocrine treatment (Exposure) utilisation (Outcome) stratified by Indigenous status (comparator).

### Indigenous terminology

Indigenous nations and tribes are diverse and have unique cultures, languages, and histories. The included studies have used different terms to collectively refer to Indigenous nations/tribes within specific regions or countries, including American Indian, Alaska Native, Native Hawaiian, Māori, Aboriginal and Torres Strait Islander, and others. This review respectfully used the term ‘Indigenous peoples’, regardless of the country of origin, for the included studies. However, we acknowledge that not all Indigenous peoples identify with this terminology. In some cases, studies have reported outcomes for Indigenous people combined with a non-Indigenous minority population (e.g., Asian/Pacific Islanders). In such cases, they werereferred to as “Indigenous/Other peoples”. Data for non-Indigenous populations were also extracted and referred in this review as “non-Indigenous Populations”. In the included studies, this included sub-group populations of anyone who was not identified as an Indigenous or Tribal person or may refer to the dominate race/ethnic group (e.g., European New Zealanders, non-Hispanic White Americans).

### Search strategy

Relevant articles were searched on PubMed, CINAHL, Web of Science, Embase and Scopus. The search terms were developed through discussing with the research team and informed by previous relevant review articles, such as the global Indigenous people search terms from Lee and colleagues [46]. The search terms were related to Indigenous peoples, endocrine or hormonal treatment, and all cancer. An example of search terms/strategies using the PubMed and CINAHL databases are presented in the supplementary files (see Additional file 2). The search was limited to articles published in English from 1973, when ETofficially began [14], until 6 February 2023. Study authors were contacted via email to get additional information and studies.

### Eligibility criteria

This review included peer-reviewed quantitative articles that reported ET use for cancer treatment for Indigenous peoples. In this review, we defined ET use based on whether the articles reported the number or percentage/proportion of prescribed ET use for cancer, noting it as ‘Yes’ if ET for cancer had been initiated among Indigenous peoples. ET options included estrogen receptor modulators, aromatase inhibitors, androgen deprivation therapy and gonadotropin-releasing hormone agonists if used as a first-line or adjuvant cancer treatment.

**Fig. 1:**
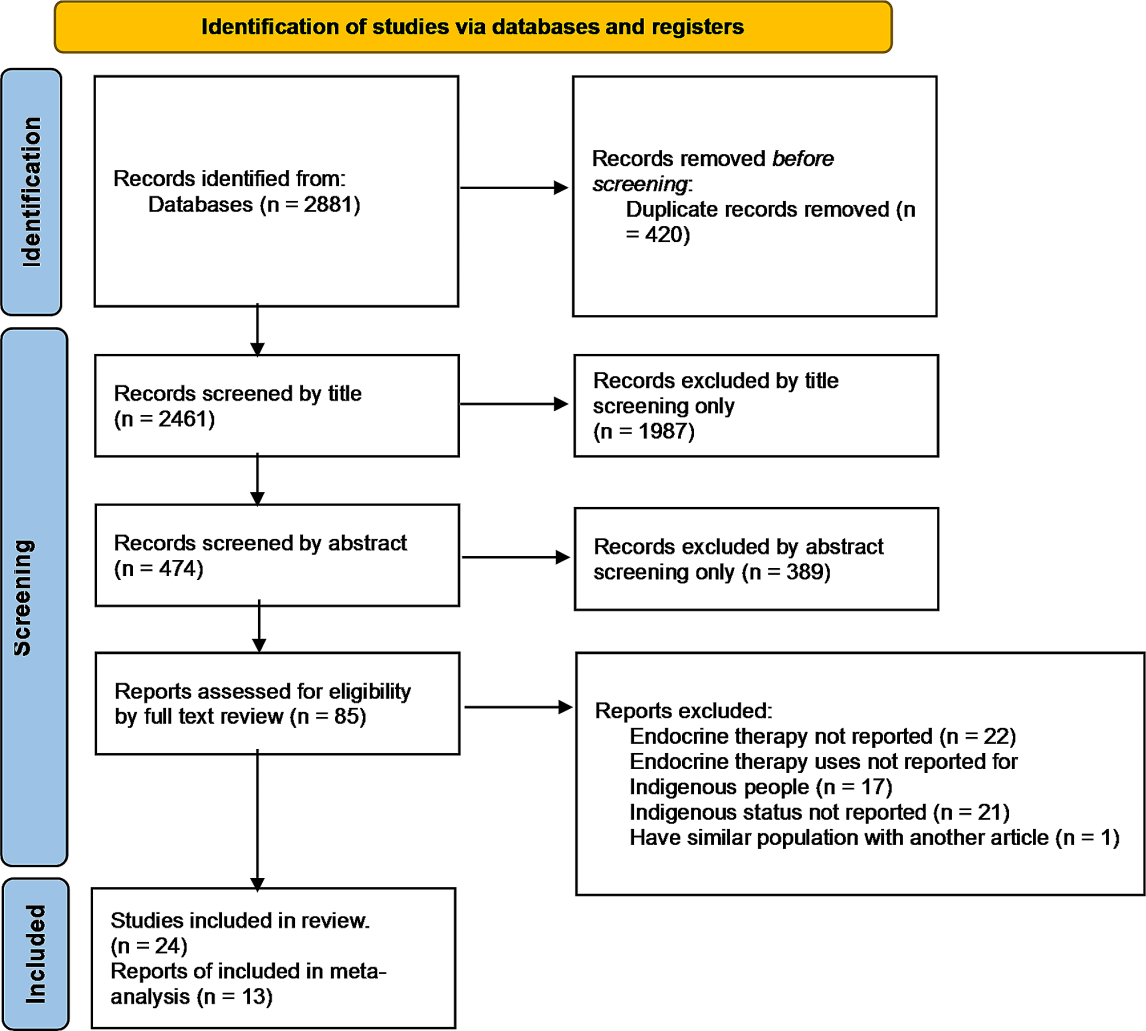
PRISMA flow diagram for study selection

Data on factors linked with ET use were extracted from the eligible articles if reported by ethnicity (Fig. 1). Risk factors for ET use were identified by reviewing the relevant literature [15, 17–29] and discussing it with the research team. Relevant risk factors were presented using the domains of the socioecological theoretical framework, which considers the influence of individual, interpersonal, and community/organisational factors and is widely used in cancer treatment research, including ET [15, 29, 47, 48]. Each factor could be linked with ET use directly or indirectly through the complex and interacting relationship [15, 29, 47, 48]. Reviews, book chapters, editorial letters, commentaries, meeting notes, opinion papers, and mass-media publications were excluded.

### Study selection

Initially, duplicate articles that were identified through Endnote Software’s automatic checking system [49] and/or through the manual search were removed. The remaining articles were exported to Rayyan Online Software [50] for title, abstract and full-text screening by two independent reviewers (SAB and HMB). Any discrepancies were resolved through discussions between the reviewers. In the full-text review, the reasons for exclusion were noted (Fig. 2). It is important to note that automatic screening methodology was not used for this review.

### Critical appraisal

The quality of the included articles for final review was assessed using the Joanna Briggs Institute’s critical appraisal checklist for prevalence studies [51]. Articles were evaluated independently by two reviewers (SAB and HMB). The critical appraisal tool has nine domains, including but not limited to the appropriateness of sampling frame and sampling, whether the sample size and response rate were adequate, and whether the statistical analysis was appropriate.

### Data extraction

Two reviewers (SAB and HMB) performed the data extraction using a customised Microsoft Excel spreadsheet, and any discrepancies were addressed through discussion in order to reach consensus. The data extraction process was guided by headings, including study characteristics (author name, year of publication, study period, study country, data source, and sample size), endocrine treatment characteristics (primary or adjuvant treatment, drug name); outcome measures such as proportion and/or effect measures (odds ratio, relative risk) and the socioecological risk factors of ET use.

### Data analysis and synthesis

The estimated measures in the articles with unknown race/ethnicity groups were excluded as they could include both Indigenous and non-Indigenous peoples in one group and would be less informative. Using the freely available WordClouds software (https://www.wordclouds.com/), ET/drug names in the included articles were visualised, where the larger the font size, the more frequently the specific treatment/drug name was mentioned in the included articles. Additionally, a socioecological theoretical framework was used to identify and describe relevant factors that could impact ET use [15, 29, 47, 48].

A meta-analysis was conducted using STATA version 17.0 (Stata Statistical Software: Release 17.0 College Station, TX: StataCorp LLC) to determine the pooled estimate of proportions of ET utilisation. A random effects meta-analysis was performed for all relevant articles to estimate pooled proportions of ET utilisation with a 95% confidence interval [37–39]. To evaluate the heterogeneity of articles, the I-squared statistics was primarily utilised along with X^2^ and Tau. To investigate the potential source of heterogeneity, sub-group and meta-regression analysis were performed. The moderators that were considered included the geographical location of the studies, primary cancer site or type, hormone receptor status, invasiveness of cancer, sample size, treatment intent and sex of the participants, and year of publication. Funnel plot, along with Egger’s test, was used to identify any potential publication bias [52–54]. Sensitivity analysis was conducted by excluding individual articles to explore the potential effect of outliers on the overall estimate, and after visually inspecting the results, studies that had a discernible influence on the overall estimate were removed, and a new estimate was pooled without their inclusion.

## Results

### Article selection

In the systematic database search, 2881 articles were identified, of which 420 were duplicates (Fig. 1). Of the 2461 articles reviewed by title, 474 relevant articles were identified for further abstract review. Twenty-four articles were included for qualitative synthesis out of 85 articles reviewed by their full text. Nearly three quarters (*n* = 43) out of 61 excluded articles did not report either ET (*n* = 22) or Indigenous status (*n* = 21) (Fig. 2).

Of the 24 articles initially identified as eligible, two reported relative measures exclusively, while nine reported ET rates for Indigenous peoples and non-Indigenous peoples combined, such as Asian/Pacific Islander (from now onwards, referred to them as ‘Indigenous/Other’). The remaining 13 reported ET rates separately for Indigenous peoples. These 13 articles were included in the meta-analysis as the primary focus of this review was to estimate the rate of ET utilisation among Indigenous cancer patients. The aforementioned non-Indigenous populations were used as the main non-Indigenous comparator, which was selected because this approach is aligned with previous studies [24, 36, 55], and their sample size was significantly larger than the other non-Indigenous groups, which might be attributed to their dominant and non-marginalised status resulting in more comprehensive data availability. However, the pooled estimate of ET use for the rest of the populations is also provided as an additional file.

### Characteristics

Nearly two million participants (*N* = 1,830,251) were included in this review. Specifically, 12,933 participants were separately reported as Indigenous (identified in 13 articles), 5,132 participants were reported as Indigenous/Other noted in 9 of the articles, and the remaining 1,812,186 participants were all non-Indigenous, including (1,625,414 Whites/Europeans and 186,772 other races/ethnicities, including Black, African American, Asian, Latino) (Table 1).

**Table 1:** The characteristics of included articles, sorted by publication year

ID	Study period	Study area^1^	Data source^3^	Population^3^	Sample size^4^	Endocrine therapy use (%)	Associations with endocrine use (95%CI)^5^
Engelking, 2023 [57]	2004–2017	USA (national)	HCR	Women aged ≥ 18 years who diagnosed with stage I–III estrogen receptor‑positive breast cancer	771,619 (2473 American Indian/Alaskan Native and 769,146 non-Hispanic White)	95 American Indian/Alaskan Native and 95 non-Hispanic White) (*P* = 0.67)	Compared to non-Hispanic Whites, 0.945(0.791–1.13) for American Indian/Alaskan Native ^a1^
Champion, 2022 [60]	2004–2014	USA (national)	HCR	Women aged ≥ 18 years and have lumpectomy or mastectomy for stage 0-IV breast cancer	903,008 (2398 American Indian, 768,396 White, 98,217 Black, 33,997 other)	63.8 American Indian, 65.2 White, 53.9 Black, 64.6 other	Not reported
Wallner, 2022 [76]	2009–2017	USA (sub-state)	PCR, census	Women aged ≥ 18 years and newly diagnosed with stage IV breast cancer	995 (110 API, 26 other/Mixed/Native American, 522 White, 139 African American, 198 Hispanic)	44.6 API, 50 other/Mixed/Native American, 51.3 White, 41.7 African American, 51.1 Hispanic *(P =* 0.260*)*	Not reported
Emerson, 2021 [31]	1997–2014	USA (sub-state)	PCR, PCD, MR, census	Women aged ≤ 49 years who diagnosed with stage I-III hormone receptor-positive breast cancer	23,680 (98 American/Indian/Alaskan Native, 3066 API, 16,677 White, 2355 Hispanic, 1484 non-Hispanic black)	78.6 American/Indian/Alaskan Native, 84.7 API, 82.5 White, 83.0 Hispanic, 78.0 non-Hispanic black	Compared to non-Hispanic Whites, 0.76 (0.46–1.24) for AMERICAN INDIAN/ALASKAN NATIVE s and 1.04 (0.93–1.16) for APIs ^a2^
Fukui, 2021 [77]	2003–2017	USA (state)	PCR, MR, survey	Women aged ≥ 54 years and had breast cancer surgery	379 (118 Native Hawaiian/Pacific Islander, 129 White, and 132 Asian)	35.6 Native Hawaiian/Pacific Islander, 25.6 White, and 32.6 Asian	Not reported
Bandera, 2020 [58]	2000–2012	USA (national)	HCR	Young adult women (< 40 years) who diagnosed with lobular carcinoma in situ	749 (61 API, 497 White non-Hispanic, 56 White Hispanic, 116 African American, 19 other)	73.8 API, 78.3 White non-Hispanic, 89.3 White Hispanic, 85.3 African American, 78.9 other (*P = 0.11)*	Not reported
Nahleh, 2020 [78]	2010–2015	US (national)	HCR, MR, census	Women and Men diagnosed with stage I luminal HER2-positive breast cancer	37,523 (100 Native American, 29,867 White, 3834 African American, 1542 Asian, 2000 Hispanic/Latinos, 180 others)	87 Native American, 86.6 White, 85.2 African American, 85.9 Asian, 83.1 Hispanic/Latinos, 82.7 others	Not reported
Nunes, 2019 [79]	2008–2015	USA (national)	MR	Women aged > 40 years who diagnosed with breast cancer and registered in MR	1864 (25 API, 1620 non-Hispanic White, 140 non-Hispanic Black, 40 Hispanic, 39 others)	68 API, 70.7 non-Hispanic White, 73.6 non-Hispanic Black, 80 Hispanic, 79.4 others	Not reported
Tin Tin, 2018 [59]	2000–2014	NZ (state)	PCR, AD	Women diagnosed with primary invasive breast cancer	13,670 (1283 Maori, 898 Pacific and 11,489)	67.2 Maori, 65.7 Pacific and 62.9 non-Maori/Pacific (*P < 0.001)*	Not reported
Blackmore, 2018 [80]	2000–2013	NZ (state)	PCR, AD	Women aged ≥ 70 years and diagnosed with stage I–IV breast cancer	2640 (125 Maori, 98 Pacific, 2276 European, 141 other)	Not reported	Compared to others, Maori was 2.71(1.10–6.69)
Voci, 2018 [25]	2000–2012	USA (national)	HCR	Women aged 15–39 years and underwent breast conservation therapy after diagnosis of ductal carcinoma in situ (stage 0)	1795 (122 API, 1159 White, 340 Black, 130 Hispanic, 44 other)	74.6 API, 82.6 White, 82.4 Black, 91.5 Hispanic, 84.1 other (*P = 0.0123)*	Compared to Whites, 0.65(0.419–1.013) for API was ^a3^
Lawrenson, 2017 [81]	2000–2013	NZ (state)	PCR, AD	Women diagnosed with stage I–III breast cancer	9015 (891 Maori, 548 Pacific and 7576 others)	68.2 Maori, 60.4 Pacific and 60.7 others	Compared to others, 1.21(0.94–1.56) for Maori and 0.81(0.59–1.12) for Pacific in the estrogen/progesterone-positive and HER2-negative group ^a4^
Karunasinghe, 2016 [34]	2013–2014	NZ (state)	MR, AD, Survey	Men diagnosed with prostate cancer and registered in the hospital administrative database	206 (14 Maori/Pacific/East Asian, 192 Caucasian)	50 Maori/Pacific/East Asian, 35.4 Caucasian	Not reported
Lawrenson, 2015 [32]	2009–2012	NZ (state)	PCR, PCD	Men diagnosed with metastatic prostate cancer	234 (26 Maori/Pacific and 208 non-Maori/Pacific)	80.8 Maori/Pacific and 83.2 non-Maori/Pacific	Compared to Maori/Pacific, the RR was 1.14 (0.49–2.66) for non-Maori/Pacific
Seneviratne, 2015 [82]	1999–2012	NZ (state)	PCR, AD	Women aged 45–69 years and had more than one episode of invasive breast cancer	2679 (419 Maori and 2,260 European)	67.1 Maori and 71.6 European (*P = 0.058)*	Not reported
Wang, 2015 [83]	2004–2012	NZ (national)	PCR, PCD, AD	Men aged ≥ 40 years and diagnosed with prostate cancer	23,401 (1345 Maori, 649 Pacific, 20,844 European, 468 Asian, 95 other)	44.8 Maori, 46.7 Pacific, 35.5 European, 39.5 Asian, 35.8 other	Not reported
Keegan, 2015 [55]	2004–2007	USA (sub-state)	PCR, MR, AD, census	Women newly diagnosed with invasive breast cancer and have uniform access to health care and treatment	5945 (808 API, 4047 non-Hispanic White, 460 non-Hispanic African American, 630 Hispanic)	46.9 API, 52 non-Hispanic White, 36.5 non-Hispanic African American, 41.4 Hispanic	Not reported
Lawrenson, 2014 [84]	2006–2011	NZ (national)	PCR, PCD	Men diagnosed with prostate cancer	15,947 (908 Maori, 445 Pacific and 14,594 non-Maori/Pacific)	38.5 Maori, 38.9 Pacific and 30.5 non-Maori/Pacific	Compared to non-Maori/Pacific, 2.05(1.43–2.94) for Māori and 3.14(1.87–5.27) for Pacific ^a5^
Bailes, 2013 [30]	1996–2009	USA (sub-state)	HCR	Women aged ≥ 18 years who diagnosed with ductal carcinoma in situ and underwent breast-conserving therapy	1131 (62 API, 827 White, 134 African American, 108 Hispanic)	62.9 API, 39.2 White, 50.8 African American, 49.1 Hispanic (*P < 0.001)*	Not reported
Wu, 2012 [26]	2004	US (state)	PCR, MR, census	Women aged ≥ 20 years and diagnosed with locoregional breast cancer	6734 (58 Non-Hispanic American Indian/Alaskan Native, 373 API, 3907 White, 1811 Black, 585 Hispanic)	87.9 Non-Hispanic American Indian/Alaskan Native, 73.6 API, 80.3 White, 80.9 Black, 76.2 Hispanic (*p = 0.014)*	Compared to White, 1.46(0.57–3.69) for Non-Hispanic American Indian/Alaskan Native and 1.21(0.84–1.73) for API ^a6^
Haque, 2010 [85]	1990–2001	US (sub-state)	PCR, MR, census	Women aged 20–84 years and treated with breast-conserving therapy after diagnosis of ductal carcinoma in situ	3000 (370 API, 2075 Non-Hispanic White, 292 Black, 263 Hispanic)	19.5 API, 14.8 non-Hispanic White, 13 Black, 18.3 Hispanic (*p = 0.10)*	Compared to non-Hispanic Whites, Tamoxifen use was 0.87 (0.67–1.13) for API ^a7^
Enger, 2006 [33]	1990–1994	USA (state)	PCR, MR, AD	Women aged ≥ 65 years and diagnosed with stage I-II breast cancer for first-time	1859 (53 API, 1523 non-Hispanic White, 93 Hispanic, 190 African American)	75.5 API, 65.4 non-Hispanic White, 74.2 Hispanic, 61.6 African American (*P = 0.08)*	Compared to non-Hispanic Whites, no tamoxifen prescription for APIs was 0.8(0.4–1.6) ^a8^
Issell, 2005 [35]	1995–1996	US (state)	PCR, MR, survey	Women newly diagnosed with breast cancer	406 (53 Hawaiian, 98 Caucasian, 149 Japanese, 28 Filipino, 46 Chinese, 32 other)	52.8 Hawaiian, 52 Caucasian, 59.1 Japanese, 46.4 Filipino, 50 Chinese, 53.1 other (*p > 0.05)*	Not reported
Prehn, 2002 [36]	1994	US (sub-state)	PCR, MR, census	women aged ≥ 50 years and diagnosed with estrogen receptor-positive localised breast carcinoma	1772 (42 API, 1556 White, 73 Chinese, 32 Japanese, 69 Filipino)	Not reported	Compared to White, not using endocrine therapy among APIs was 0.9(0.3–2.7) ^a9^

^1^ the study conducted at the sub-national level was grouped either state (one or more administrative units i.e., states, regional administrative units) or sub-state (locations within the state or regional administration unit)

^2^ list of data sources reported in the article

^3^ the age group of the population was included if reported by article; the status of sex is reported in the population

^4^ the sample size did not include the unknown race categories due to the study focused on known race/ethnicity categories (i.e. unknown race/ethnicity group could include both Indigenous and/or Whites/Europeans/Others)

^5^ unless specified the associations (relative measures) was in the odds ratio for endocrine treatment use. Only one relative risk was reported as in Lawrenson, 2015 [32]’ row

^a1^ the odds ratio adjusted for area, age at diagnosis, year of diagnosis, tumor grade, lymph node status, tumor size, progesterone receptor status, HER2 receptor status, and insurance

^a2^ the odds ratio adjusted for the stage at diagnosis, year of diagnosis, Charlson Comorbidity Index, education, and income

^a3^ the odds ratio adjusted for age and income

^a4^ the odds ratio adjusted for age, year of diagnosis, cancer stage, cancer grade and Charlson Comorbidity Index

^a5^ the odds ratio adjusted for age, ethnicity, year of diagnosis and orchidectomy

^a6^ the odds ratio adjusted for age, registry, and clinical variables (i.e., lymph node, histology, tumor size, grade, estrogen/progesterone receptor status, and comorbidity), insurance, census-tract poverty, census-tract education, and cancer-accredited hospital status

^a7^ the odds ratio adjusted for age, year of diagnosis, family history of breast cancer, education, income, history of diabetes, body mass index, method of cancer detection

^a8^ the odds ratio adjusted for age, Charlson Comorbidity Index, and risk of recurrence

^a9^ the odds ratio adjusted for tumor size, and histologic subtype, grade and membership in health maintenance organisation

AD = Administrative Database (e.g. administrative and clinical database during service use e.g. outpatient visit, admission, discharge), API = Asian/Pacific Islander, HCR = Hospital-based Cancer Registry, HER2 = Human Epidermal growth factor Receptor 2, MR = Medical Records, NZ = New Zealand, PCD = Pharmaceutical Collection Database, PCR = Population-based Cancer Registry, USA = the United states of America

More than 80% (*n* = 20) of the articles examined breast cancer, with two-thirds examined invasive cancers only (*n* = 13), and the remainder examined preinvasive (*n* = 4) or both preinvasive and invasive breast cancer types (*n* = 3). Four prostate cancer types were reported (three invasive and one both preinvasive and invasive) (Table 1). Of the 18 articles that reported cancer hormone receptor status, five were based on all hormone-positive cancers, while the rest (*n* = 13) were based on ≥ 75% hormone receptor cancers (*n* = 5) or 60–75% hormone receptor cancers (*n* = 8). Of 10 articles that reported the HER2 (human epidermal growth factor receptor 2) status, one article included all HER2-positive cancers, while the remaining articles (*n* = 9) had 10–19% HER2-positive cancers (*n* = 6) or 5–10% HER2-positive cancers (*n* = 3).

All articles used an administrative database for cancer diagnosis, primarily cancer registries (*n* = 22), of which approximately two-thirds were population-based cancer registries (*n* = 15). More than half (*n* = 13) of the articles reported using a pharmaceutical collection database (*n* = 4) or reviewed medical records (*n* = 9) for ET data. Three quarters of the articles (*n* = 17) reported the use of one or more of the following data sources for sociodemographic factors: administrative database (*n* = 9), medical record (*n* = 8), census tract data (*n* = 7) or surveys (*n* = 3) (Table 1).

Two third of the articles were conducted in United States (*n* = 16), and the rest were conducted in New Zealand (*n* = 8). Sixteen studies were conducted at the sub-national level. The median reported study period was 9.3 years, with half (*n* = 12) covering more than ten years and the rest covering 5–10 years (*n* = 6) or ≤ 5 years (*n* = 6) (Table 1).

Regarding treatment/drug names, half of the articles (*n* = 12) used hormones, while the rest reported specific drug names (the commonly reported ones were tamoxifen, aromatase inhibitors, androgen deprivation therapy (ADT) and anti-androgens and luteinising hormone-releasing hormone (LHRH) analogues). Details of the reported treatment/drug names are presented in the supplementary file (see Additional file 3). Sixteen out of 18 articles reported using ET as an adjuvant treatment option, and the remaining two reported its use for both primary and adjuvant treatment.

### Pooled estimates of the use of endocrine treatment for cancer

The ET utilisation rate was reported in 22 articles (Table 1), with 13 of these articles separately reported the usage among Indigenous peoples diagnosed with cancer. According to the 13 studies, the ET rate among Indigenous peoples was 67% (95% CI: 54–80), with significant evidence of between-studies heterogeneity (X^2^ = 3737.1, I^2^ = 99.6%, *P* < 0.001), rates ranging from 36 to 95% (Fig. 2a). In the 13 studies, the pooled ET use estimate among non-Indigenous populations was 67% (95% CI:53–81) (Fig. 2b). Additional pooled estimates of ET use are provided in the supplementary file (all provided within Additional file 4): the estimate for Indigenous/Other (*n* = 9 studies) is provided in Figure S1 within Additional file 4, the pooled estimate for ‘Indigenous alone and Indigenous/Other’ is presented in Figure S2 within Additional file 4, and the estimate for all non-Indigenous peoples in the 22 articles is provided in Figure S3 OF within Additional file 4.

**Fig. 2a:**
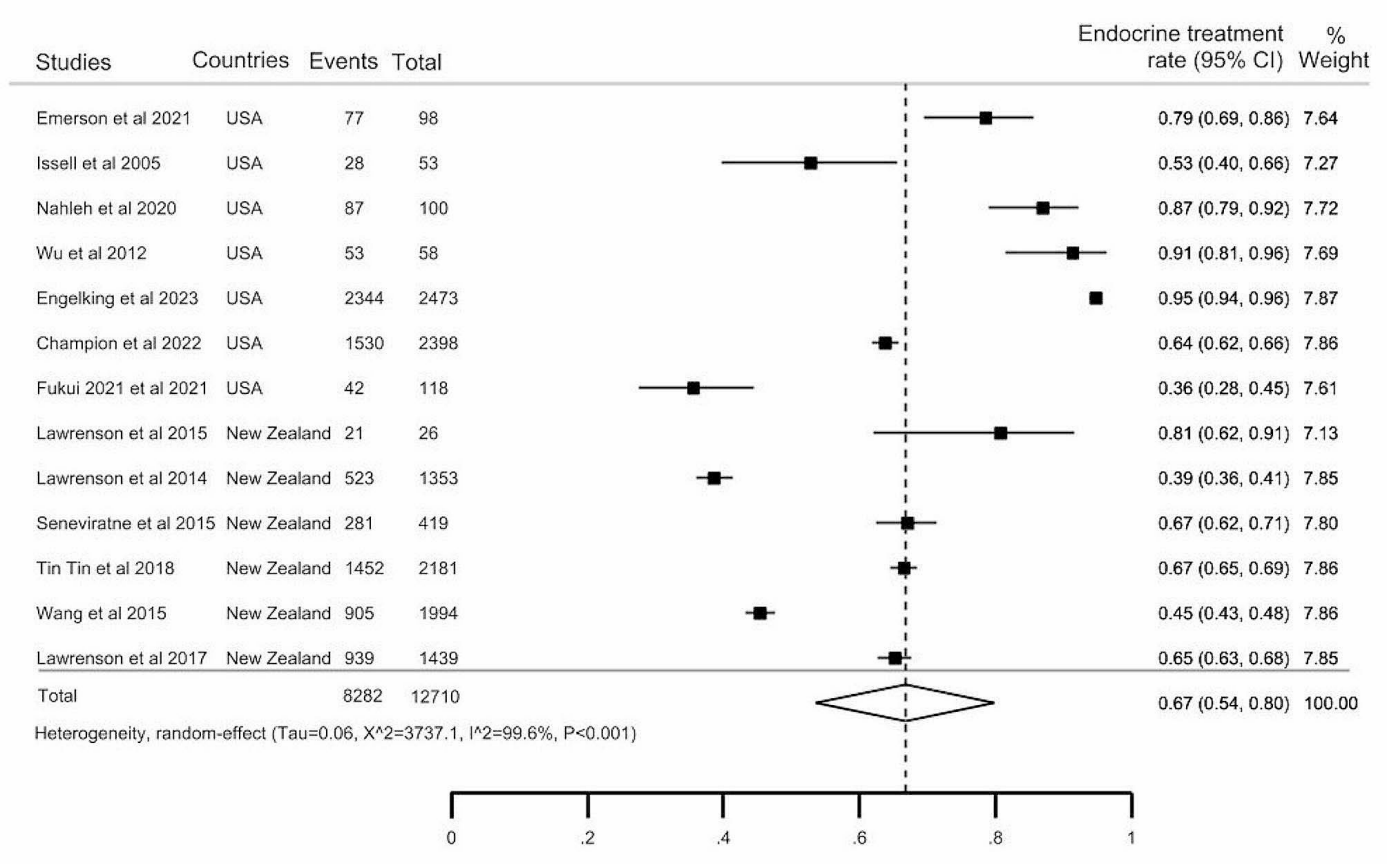
The pooled proportion of endocrine therapy utilisation among the Indigenous people diagnosed with cancer, as reported in 13 articles

**Fig. 2b:**
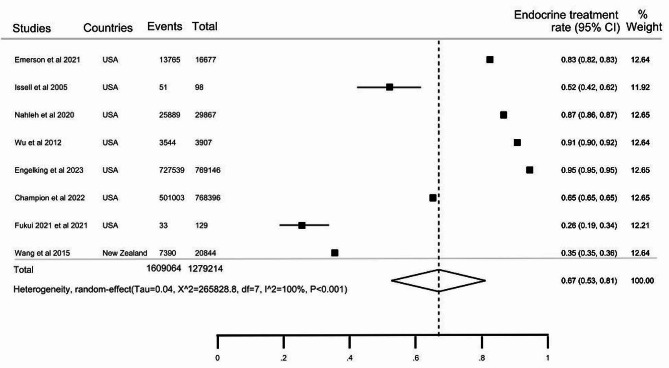
The pooled proportion of endocrine therapy use among non-Indigenous populations diagnosed with cancer as reported in the articles (of the 13 articles)

### Sub-group analysis and meta-regression

The ET rates in Indigenous peoples in the USA and New Zealand were 72% (95% CI: 56–88) and 60% (95% CI: 49–71), respectively (Table 2). The rate of treatment among Indigenous peoples diagnosed with breast cancer was 71% (95% CI: 59–82), while it was 50% (95% CI: 40–59) for prostate cancer. The pooled estimated proportion of ET in articles with less than 500 participants was 71% (95% CI: 57–84) and 62% (95% CI: 43–82) in articles with more than 500 individuals. Due to the high level of heterogeneity observed between articles, we conducted a meta-regression to investigate the potential sources of this variation. From the sources of variation examined in the meta-regression, we identified four potential moderators: geographical location, cancer type, hormone receptor status, and cancer invasiveness, with a *P* ≤ 0.2 in the univariable analysis [56]. These covariates were included in the multivariable model, which yielded a significant model (*P* = 0.012) and explained 74.7% of the between-study variation.

**Table 2:** Sub-group analysis of the rate of endocrine therapy utilisation − 13 articles reporting the rate in indigenous peoples

Sub-group variables	Endocrine utilisation rate, %	95% Confidence intervals	Heterogeneity (I^2^)	Number of articles
**Total**	67	54–80	99.6%, *P* < 0.001	13
**Geographical location**				
USA	72	56–88	99.4%, *P* < 0.001	**7**
New Zealand	60	49–71	98.9%, *P* < 0.001	6
**Primary cancer site**				
Breast	71	59–82	99.4%, *P* < 0.001	10
Prostate	50	40–59	Not applicable	3
**Sex**				
Female	69	66–81	99.5%, *P* < 0.001	9
Male	50	40–59	Not applicable	3
Female and Male	87	79–92	Not applicable	1
**Invasiveness of the cancer**				
Invasive	**77**	64–89	99.3%, *P* < 0.001	9
Invasive/non-invasive*	50	36–63	99.2%, *P* < 0.001	4
**Treatment intent** ^**a**^				
Adjuvant	75	60–89	99.3%, *P* < 0.001	8
Adjuvant/primary treatment**	57	56–58	Not applicable	2
**Data record**				
2000 or earlier***	67	64–70	70.3%, *P =* 0.01	5
After 2000 only	70	51–88	99.7%, *P* < 0.001	8
**Sample size**				
Less than 500	71	57–84	95.4%, *P* < 0.001	7
More than 500	62	43–82	99.8%, *P* < 0.001	6
**Hormone receptor status** ^**a**^				
All participants hormone positive	87	78–97	Not applicable	3
Some participants hormone negative	64	59–70	94.2%, *P* < 0.001	7
**Publication year** ^**b**^				
2017-recent	70	56–85	99.6%, *P* < 0.001	7
Before 2017	62	49–75	98.2%, *P* < 0.001	6

*Articles that included both invasive and non-invasive cancer patients **Articles that reported endocrine use as primary treatment and adjuvant. ***Articles that included patient data from the year 2000 or earlier ^a^Three articles did not report receptor status ^b^Published within the last 5 years or before.

### Sensitivity analysis and publication bias

After excluding five articles with outlier values, the pooled estimate of ET use among Indigenous peoples decreased from 70 to 67%, while the pooled estimate among non-Indigenous populations increased from 67 to 72% when four articles with outlier values were excluded (see Additional file 5). Furthermore, the funnel plot used to examine publication bias in the thirteen studies included in the pooled estimation revealed no significant evidence of publication bias, as the data points were evenly distributed (see Additional file 6). The Egger test *(P* = 0.11) also yielded a non-significant result, further supporting our conclusion that there was no publication bias.

### Quality appraisal

Nine articles met all nine criteria on the JBI’s critical appraisal checklist for prevalence studies, while the remaining 15 articles fulfilled eight (*n* = 13) or seven (*n* = 2) of the checklists. All studies were carried out using population-based data. The overall sample size was large in most studies (around 1000 and above in 20 articles), while the subpopulation level sample size by ethnic group was smaller in Indigenous peoples. The appraisal of each article is presented in the supplementary material (see Additional file 7).

### Qualitative synthesis of relevant factors that may impact ET use

**Fig. 3:**
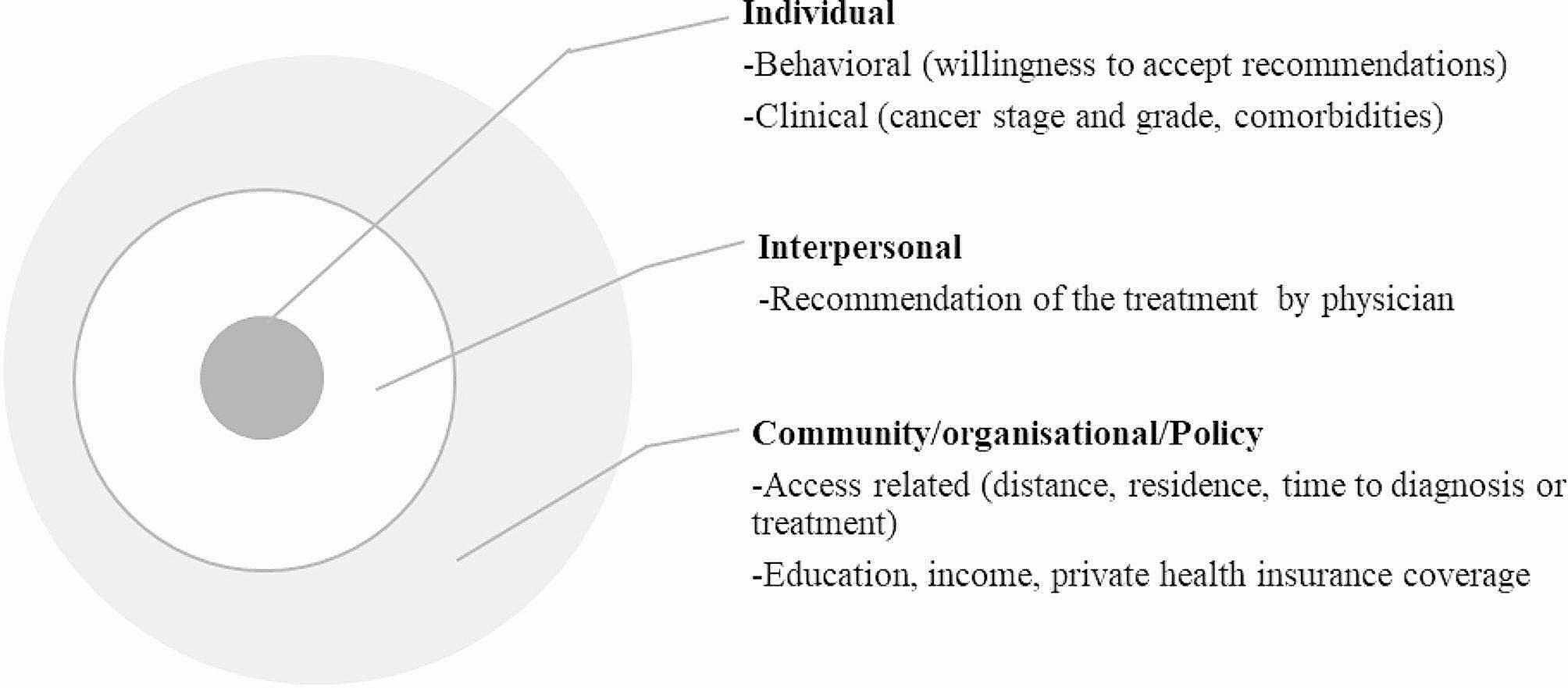
The socioecological framework guiding description of factors related with endocrine treatment use

Seventeen articles described and/or compared one or more factors (ranging from one to ten factors) that could affect the ET use by Indigenous status (Table 3). The section below focuses on presenting a summary of some of the relevant factors related to ET use among Indigenous people in comparison to non-Indigenous populations [24, 36, 55]. The description of these factors among non-Indigenous populations and other minority races/ethnicities (African American or Hispanic/Latinos) are also presented in Table 3. In addition, Fig. 3 illustrates factors that may influence the ET use against the relevant domains of the socio-ecological model.

**Table 3:** The summary of the distribution of the risk factors of endocrine treatment use by indigenous status, sorted by reporting many factors and publication year

ID	Age in years (%)^1^	Stage of cancer (%)^2^	Grade of cancer (%)		Comorbidity (%)^3^	Socioeconomic factors (%)^4^	Access, behavioural and other factors (%)^5^	
Champion, 2022 [60]	Mean age: 54 American Indian, 58 White, 57 Black, 54 others	Metastasised: 2.0 American Indian, 1.2 White, 1.9 Black, 1.3 other		Grade III: 36.0 American Indian, 30.0 White, 45.6 Black, 35.5 other.	CCI 1+: 24.2 American Indian, 15.0 White, 21.6 Black, 12.5 other	• Income group <$48,000: 54.8 American Indian, 32.6 White, 60.3 Black, 18.8 other • High school graduation rate ≤ 87%: 52.3 American Indian, 33.7 White, 65.3 Black, 36.5 other • Having private health insurance: 40.5 American Indian, 54.1 White, 50.3 Black, 62.9 other		• Median distance travelled to a treatment facility in miles: 13.1 American Indian, 8.1 White, 5.4 Black, 6.8 other • Time to surgery (median in days): 44 American Indian, 39.5 White, 47 Black, 41.5 other • BMI ≥ 25: 48.2 API, 58.0 other/Mixed/Native American, 61.5 White, 69.1 African American, 70.2 Hispanic *(P* < 0.0001*)*
Wallner, 2022 [76]	Age group < 50: 32.7 API, 23.1 other/Mixed/Native American, 17.1 White, 23.0 African American, 30.8 Hispanic *(P = <* 0.0001*)*	All stage IV		Grade III: 53.4 API, 47.8 other/Mixed/Native American, 44.2 White, 43.2 African American, 46.5 Hispanic *(P* = 0.511*)*	• Elixhauser comorbidity index 3+: 60 API, 23.1 other/Mixed/Native American, 39.8 White, 54.7 African American, 33.3 Hispanic *(P* < 0.0001*)* • Depression: 4.6 API, 7.7 Other/Mixed/Native American, 19.4 White, 15.8 African American, 12.6 Hispanic *(P* < 0.0001*)*	Median annual household income ≤$65,000: 48.2 API, 69.2 other/Mixed/Native American, 41.6 White, 75.4 African American, 60.1 Hispanic *(P* < 0.0001*)*		• BMI ≥ 25: 48.2 API, 58.0 other/Mixed/Native American, 61.5 White, 69.1 African American, 70.2 Hispanic *(P* < 0.0001*)* • Time to surgery (median in days): 31 API, 11 other/Mixed/Native American, 32 White, 36 African American, 29 Hispanic *(P* = 0.75*)*
Tin Tin, 2018 [59]	Mean age: 54.8 Maori, 54.1 Pacific and 59.6 non-Maori/Pacific (*P < 0.001)*	Stage IV: 7.6 Maori, 10.9 Pacific and 3.9 non-Maori/Pacific (*P < 0.001)*		Grade III: 27.1 Maori, 34.9 Pacific and 28.4 non-Maori/Pacific (*P < 0.001)*	CCI 2: 24.1 Maori, 23.3 Pacific and 11.9 non-Maori/Pacific (*P < 0.001)*	Live in the most deprived neighbourhood (scale 9–10): 45.1 Maori, 53.5 Pacific and 13.5 non-Maori/Pacific (*P < 0.001)*		• Live in rural: 21.7 Maori, 5.1 Pacific and 13.6 non-Maori/Pacific (*P < 0.001)* • Private hospital use: 13.4 Maori, 12.7 Pacific and 47.4 non-Maori/Pacific (*P < 0.001)* • Time to first treatment (Mean in days): 41.5 Maori, 62.1 Pacific and 30.3 non-Maori/Pacific (*P* < 0.001) • Non screen diagnosed cancer: 65 Maori, 70.1 Pacific and 60.5 non-Maori/Pacific (*P < 0.001)*
Seneviratne, 2015 [82]	Mean age: 55.6 Maori and 61.4 European (*P < 0.001)*	Stage IV: 11.0 Maori and 4.6 European (*P < 0.0067)*		Grade III: 24.1 Maori and 22.9 European (*P < 0.001)*	CCI 3+: 3.3 Maori and 1.5 European (*P < 0.001)*	Live in the most deprived neighbourhood (scale 9–10): 40.2 Maori and 22.8 European (*P < 0.001)*		• Live in semi-urban: 34.6 Maori and 27.2 European (*P < 0.008)* • Private hospital use: 9.3 Maori and 31.9 European (*P < 0.001)* • Non screen diagnosed cancer: 70.2 Maori and 63.1 European (*P < 0.006)* • Delayed surgery (> 60 days): 18.5 Maori and 11.7 European (*P < 0.006)* • BMI > 30: 52.4 Maori and 28.1 European (*P < 0.001)* • Current smoker: 46.1 Maori and 15.2 European (*P < 0.001)*
Haque, 2010 [85]	Age < 50 years: 37.6 API, 22 non-Hispanic White, 27.7 Black, 31.9 Hispanic (*p < 0.001)*	Tumor size ≥ 2 cm: 24.5 API, 22.4 non-Hispanic White, 38.7 Black, 31.7 Hispanic (*p = 0.005)*		High nuclear grade: 41.1 API, 41.5 non-Hispanic White, 42.6 Black, 42.5 Hispanic (*p = 0.005)*	History of diabetes: 8.1 API, 6.1 non-Hispanic White, 11.7 Black, 11.4 Hispanic (*p < 0.001)*	• Live in a local area with a median family income ≤$60,438: 35.7 API, 34.0 non-Hispanic White, 58.6 Black, 54.4 Hispanic (*p < 0.001)* • High school graduate group: 19 API, 17.3 non-Hispanic White, 48.9 Black, 45.4 Hispanic (*p < 0.001)*		Overweight/obese: 33.5 API, 52.0 non-Hispanic White, 69.5 Black, 66.9 Hispanic (*p < 0.001)*
Engelking, 2023 [57]	Age group 18–44: 13 American Indian/Alaskan Native and 10 non-Hispanic White) (*P < 0.0001*)	Stage III: 23 American Indian/Alaskan Native and 20 non-Hispanic White) (*P < 0.0001*)		Grade III/IV: 23.4 American Indian/Alaskan Native and 19.8 non-Hispanic White) (*P < 0.0001*)	CCI 1+: 25 American Indian/Alaskan Native and 15 non-Hispanic White) (*P < 0.0001*)	Insured by private insurance: 98 American Indian/Alaskan Native and 99 non-Hispanic White) (*P < 0.0001*)		Live in rural: 8 American Indian/Alaskan Native and 2 non-Hispanic White) (*P < 0.0001*)
Emerson, 2021 [31]	Age group ≤ 49: 27.3 American Indian/Alaskan Native, 30.9 API, 14.5 White, 27.8 Hispanic, 22.6 non-Hispanic Black	Regional: 30.6 American Indian/Alaskan Native, 30.4 API, 29.1 White, 34.6 Hispanic, 33.8 non-Hispanic Black		Grade III/IV: 20.4 American Indian/Alaskan Native, 22.3 API, 16.2 White, 21.7 Hispanic, 24.5 non-Hispanic Black	CCI 2+: 22.5 American Indian/Alaskan Native, 10.1 API, 12.1 White, 13.5 Hispanic, 18.3 non-Hispanic Black	• Live in census tract with average income group <$50,000: 35.7 American Indian/Alaskan Native, 11.7 API, 17.4 White, 24.9 Hispanic, 40.2 non-Hispanic Black. • Live in census tracts with less than high school completion (≥ 10%): 23.5 American Indian/Alaskan Native, 23.0 API, 14.8 White, 36.3 Hispanic, 35.3 non-Hispanic Black		Not reported by race/ethnicity
Lawrenson, 2017 [81]	Age group < 50: 35.1 Maori, 43.8 Pacific, 28.8 others (*P < 0.001)*	Stage III: 19.6 Maori, 28.3 Pacific, 16.0 others (*P < 0.001)*		Grade III: 26.8 Maori, 37.4 Pacific, 29.9 others (*P < 0.001)*	CCI 2+: 19.4 Maori, 17.2 Pacific, 10.8 others *(P0.045)*	Not reported by race/ethnicity		Not reported by race/ethnicity
Prehn, 2002 [36]	Mean age: 33.0 API, 38.4 White, 38.4 Chinese, 36.4 Japanese, 32.4 Filipino (*P < 0.01)*	Mean tumor size (mm): 20.1 API, 17.3 White, 21.1 Chinese, 15.5 Japanese, 19.5 Filipino (*P = 0.19)*		Poorly/undifferentiated histologic grade: 38.1 API, 21.4 White, 27.4 Chinese, 21.9 Japanese, 24.6 Filipino (*P = 0.01)*	Not reported by race/ethnicity	• Per capita income ($): 21,900 API; 30, 900 White; 24,600 Chinese; 32,300 Japanese; 20,300 Filipino (*P = 0.01)* • Women aged ≥ 25 years with college degree: 29.4 API, 38.0 White, 32.9 Chinese, 40.6 Japanese, 24.5 Filipino (*P = 0.01)*		Not reported by race/ethnicity
Fukui, 2021 [77]	Mean age: 69.9 Native Hawaiian/Pacific Islander, 70.1 White, 73.2 Asian	Regional: 28 Native Hawaiian/Pacific Islander, 18.6 White, 20.5 Asian		Not reported by race/ethnicity	Not reported by race/ethnicity	Not reported by race/ethnicity		BMI ≥ 35: 31.4 Native Hawaiian/Pacific Islander, 10.1 White, 6.1 Asian
Keegan, 2015 [55]	Age group < 45: 18.5 API, 7.9 non-Hispanic White, 11.7 non-Hispanic African American, 17.8 Hispanic	Stage IV: 3.5 API, 2.8 non-Hispanic White, 6.1 non-Hispanic African American, 2.6 Hispanic		Not reported by race/ethnicity	CCI 2+: 7.5 API, 7.0 non-Hispanic White, 10.2 non-Hispanic African American, 7.1 Hispanic	Not reported by race/ethnicity		Not reported by race/ethnicity
Issell, 2005 [35]	Age < 50 years: 37.7 Hawaiian, 22.5 Caucasian, 19.5 Japanese, 35.7 Filipino, 30.4 Chinese, 46.9 other (*p < 0.05)*	Stage III/IV: 7.6 Hawaiian, 5.1 Caucasian, 3.4 Japanese, 17.9 Filipino, 13.1 Chinese, 6.2 other (*p > 0.05)*		Not reported by race/ethnicity	At least one comorbidity: 35.9 Hawaiian, 26.5 Caucasian, 32.9 Japanese, 28.6 Filipino, 19.6 Chinese, 15.6 other (*p > 0.05)*	Not reported by race/ethnicity		Not reported by race/ethnicity
Lawrenson, 2015 [32]	Mean age: 72 Maori/Pacific and 76 non-Maori/Pacific	Not reported by race/ethnicity		Not reported by race/ethnicity	Not reported by race/ethnicity	Not reported by race/ethnicity		PSA level (≥ 1,000 ng/ml): 22.7 Maori/Pacific and 13.8 non-Maori/Pacific
Lawrenson, 2014 [84]	Not reported by race/ethnicity	Metastatic: 8.8 Maori, 10.3 Pacific and 5.5 non-Maori/Pacific		Not reported by race/ethnicity	Not reported by race/ethnicity	Not reported by race/ethnicity		Not reported by race/ethnicity
Bailes, 2013 [30]	Median age: 52 API, 55 White, 56 African American, and 50 Hispanic (*P* < 0.001)	Not reported by race/ethnicity		Grade II/III: 85.3 API, 90.8 White, 87.6 African American, 90.1 Hispanic (*P =* 0.472)	Not reported by race/ethnicity	Not reported by race/ethnicity		Not reported by race/ethnicity
Bandera, 2020 [58]	Not reported by race/ethnicity	Not reported by race/ethnicity		Not reported by race/ethnicity	Not reported by race/ethnicity	Not reported by race/ethnicity		• Physician non-Recommendation of ET: 51.2 API, 54.5 White Hispanic, 47.2 African American, 56.1 White non-Hispanic, 62.7 Other (*P = 0.1)* • Patient refused recommended ET: 26.2 API, 10.7 White Hispanic, 14.7 African American, 21.7 White non-Hispanic, 21.1 Other (*P = 0.11)*
Voci, 2018 [25]	Not reported by race/ethnicity	Not reported by race/ethnicity		Not reported by race/ethnicity	Not reported by race/ethnicity	Not reported by race/ethnicity		Physician non-Recommendation of ET: 50.6 API, 56.2 White, 50.9 Black, 54.2 Hispanic, 62.1 Other (*P = 0.0317)*

^1^ the proportions of the lowest age group categories in each race/ethnicity presented in the table if the mean age by race/ethnicity was not reported in the article

^2^ cancer stage classification was varied across articles, some reported the stage in three or more categories (e.g. stage I-IV), while others were classified as local and regional. The proportions reported in the table were for the advanced stage categories e.g. (stage III and/or IV or regional stage) whenever available

^3^ individual disease frequencies were reported in the table if the comorbidity index measure (e.g. Charlson Comorbidity Index) was not available. The proportions reported by race/ethnicity in the table were for the higher Charlson Comorbidity Index group categories

^4^ socioeconomic group includes income, socioeconomic status and educational status. The proportions reported in the table were for the lower socioeconomic status by race/ethnicity

^5^ health service access group includes area of residence, proximity to health service, private hospital use and having private health insurance, delay (delay for diagnosis or treatment). The proportions reported by race/ethnicity in the table were for delayed cancer diagnosis or treatment

Behavioural group includes smoking, and overweight (body mass index). The proportions reported by race/ethnicity in the table were for the overweight or obese body mass index group

API = Asian/Pacific Islander, BMI = Body Mass Index, CCI: Charlson Comorbidity Index, CM = Centimetre, ET = Endocrine Therapy, mm = millimetre, PSA

### Individual-level factors

In all articles (*n* = 14) that reported age by race/ethnicity, a higher proportion of Indigenous peoples were diagnosed with cancer at a younger age than non-Indigenous populations (Table 3). This age difference was statistically significant in 12 articles (reported the *P*-values and/or 95% confidence intervals of the estimates). For example, a study in New Zealand involving 2,679 women showed that Māori women were being diagnosed with breast cancer at a younger age (mean 55.6 years) compared to European women (61.4 years) (*P* < 0.001) [24]. Similarly, a study among 23,680 North American women found that the proportion of American Indian/Alaskan Native s diagnosed with breast cancer at or before 49 years of age was twice as high (27.3%) as their non-Indigenous population counterparts (14.5%) [31] (Table 3).

In all articles that reported cancer stage and/or grade by race/ethnicity (*n* = 14), a higher proportion of Indigenous peoples were diagnosed with advanced cancer stage and/or grade than non-Indigenous populations. The variation was statistically significant in six out of nine articles that reported the *p*-values and/or 95% confidence intervals of the estimates. For instance, a study conducted in New Zealand among 2,679 women revealed a higher proportion of Māori women being diagnosed with advanced breast cancer (stage IV) (11.0%) compared to European women (4.6%) (*P* = 0.007). Compared to non-Hispanic non-Indigenous populations, a higher proportion of American Indian and Alaskan Native was being diagnosed with advanced stage (stage III: 23% vs. 20%, *P* < 0.0001) or advanced grade (grade III/IV: 23.4% vs. 19.8, *P* < 0.0001) [57] (Table 3).

In ten articles that reported the comorbidity status by race/ethnicity, a higher proportion of Indigenous peoples have comorbidities when compared to non-Indigenous peoples, with significant variations observed in six out of seven articles that reported the statistical differences (Table 3). For example, a study among 771,619 women showed a higher proportion of comorbidities (one or more Charlson Comorbidity Index) in American Indian/Alaskan Native (25%) than non-Hispanic White populations (15%) (*P* < 0.0001) [57]. The proportion of comorbidities (Charlson Comorbidity Index: three or more) was also found to be higher among Māori women (3.3%) than European women (1.5%) (*P* < 0.001) [24] (Table 3).

Furthermore, based on a study carried out in the USA, it was found that the percentage of APIs (26.2%) who declined the physician’s recommendation for ET use was twice as high as that of White Hispanic populations (10.7%) (*P* = 0.11) [58] (Table 3).

### Interpersonal-level factors

Physician recommendation of ET as a cancer treatment choice enhances the utilisation of ET [25, 58]. Two articles reported the status of the physician recommendation of ET by race/ethnicity, and both indicated a slightly higher recommendation of ET for APIs compared to non-Indigenous populations e.g. 48.8% vs. 45.5% (*P* = 0.1) [58] or 49.4% vs. 43.8% (*P* = 0.03) [25] (Table 3).

### Community/organisational/policy-level factors

Hospital type and health service access-related factors (participants’ residential living area, distance to hospital, time to diagnosis or treatment) were reported by four articles, with two reported statistically significant differences *(P* < 0.05) out of the three articles that reported *p*-values. Compared to non-Indigenous populations, Indigenous peoples were more likely to live in rural areas, travel long distances to access treatment facilities, wait longer time to get diagnosis and treatment services, and were less likely to use private hospital care (Table 3). For example, a study among 771,619 women found that a four-fold higher proportion of American/Alaskan Native (8%) were residing in rural areas compared to non-Hispanic White populations (2%) (*P* < 0.0001) [57]. The proportion of Māori women residing in rural areas was also higher (21.7%) compared to non-Māori/Pacific women (13.6%) (*P* < 0.001) [59]. A study among 903,008 North American women revealed that American Indians travelled longer distances to access treatment facilities (median 13.1 miles) than White populations (8.1 miles). The proportion of Māori (13.4%) or Pacific women (12.7%) who used private hospital care was also lower compared to non-Māori/Pacific women (47.4%) (*P* < 0.001). Similarly, the mean waiting time in days from cancer diagnosis to surgery was higher among Māori (41.5) or Pacific women (62.1) than among non-Māori/Pacific women (30.3) (*P* < 0.001) [59] (Table 3).

The proportion of Indigenous and/or Indigenous/other individuals lacking private health insurance, having lower educational status, and lower income was higher compared to non-Indigenous populations. These differences were statistically significant in five out of seven articles that reported *p*-values. For instance, a study among 1,772 women with breast cancer in the USA showed that the per capita income among APIs (Asian Pacific Islanders) was lower ($21,900) compared to non-Indigenous populations ($30,900) (*P* < 0.001) [36]. The proportion of American Indians who have an annual income below $48,000 was nearly two-fold greater (54.8%) than non-Indigenous population (32.6%) [60]. Another study from USA revealed that a lower proportion of American Indians had private health insurance (40.5%) than non-Indigenous populations (54.1%) [60] (Table 3).

## Discussion

To the best of our knowledge, this is the first review assessing ET utilisation and the relevant factors that could impact its utilisation, with a focus on Indigenous peoples. This study found a global ET utilisation of 67% (95% CI:54–80) for those Indigenous people diagnosed with cancer. ET utilisation was lower among Indigenous people compared to non-Indigenous populations. In comparison to non-Indigenous populations, the Indigenous people exhibit a higher prevalence of advanced cancer or high-grade diagnoses, occurring at a younger age. Additionally, Indigenous populations experience a greater frequency of other comorbidities, are less inclined to take up-recommended treatment, receive lower income, have limited access to private health insurance and reduced utilisation of private hospital care and are more likely to live in remote areas and have extended travel distances to treatment facilities. Ensuring optimal cancer care, including appropriate ET initiation, is crucial to close the existing disparity in cancer outcomes, such as higher mortality and poor survival and quality of life, among Indigenous peoples compared to non-Indigenous peoples [4–9]. However, it seems not to be the case, according to the current review.

In light of the higher proportion of Indigenous cancer patients diagnosed with advanced cancer, the underutilisation of ET among this population (67%) raises concerns and appears incongruent with established clinical practice guidelines [61–63]. This discrepancy is particularly noteworthy considering that the guidelines, followed in various countries, including the USA and New Zealand, where the included articles were conducted, strongly advocate the use of ET for almost all hormone receptor-positive cancer cases [62, 63]. Given the guidelines, further investigation is warranted to understand the factors contributing to the observed low utilisation of ET in Indigenous peoples while also considering other potential reasons. The overall rates of ET utilisation were similar between the Indigenous and non-Indigenous populations, yet this should be interpreted with caution. For one thing, it is critical to note that this finding challenges the notion that Indigenous peoples would have a higher rate of ET prescription, considering their higher proportion of advanced cancer cases at diagnosis. It is also essential to consider the potential impact of the small sample size of the Indigenous people on accurately estimating utilisation rates. For instance, our sub-group analysis, conducted among studies with relatively larger sample size (≥ 500), revealed that Indigenous peoples had a 14% (62%; 95% CI:43 − 82) lower ET utilisation than non-Indigenous populations (76%; 95% CI:60 − 92). This finding suggests that if a larger sample size of the Indigenous people was considered when evaluating ET use, the precise estimate would likely reveal a lower rate compared to their non-Indigenous counterparts. This could delineate an existing disparity that could be attributed to limited access to treatment among Indigenous peoples as found in the studies or other factors which need further investigation.

Various factors at the level of patients, the providers and the healthcare system could lead to lower utilisation of lifesaving cancer treatments, including ET [62, 63]. For instance, evidence showed that the lower utilisation of ET among the Indigenous people could be linked with fewer recommendations of the treatment by healthcare professionals, patient refusal of the recommended ET and lack of trust in the health system due to historical systemic racism or ongoing marginalisation [15, 58, 64–66]. Some evidence highlighted the under-recommendation of ET because healthcare professionals did not perceive it as a routine breast cancer treatment option [15]. They were also 30% times less likely to suggest such treatment to patients living in remote locations [58]. A quarter of the APIs (26%) in this review refused to take the recommended ET, while the refusal rate was 10.7% among Hispanic White populations [58]. Moving forward, strengthening capacity-building training for healthcare professionals, and providing tailored health education to patients could improve ET prescription rates and patients’ drug uptake [64–66]. However, further investigation of the drivers behind the low recommendation and patient refusal are also equally important. This review also highlights the importance of addressing the factors identified to potentially influence ET service access among Indigenous peoples, including being more likely to live in remote areas, travelling long distances to health facilities and having low socioeconomic status as compared to non-Indigenous populations. The provision of tailored person-centred services, including transport and other logistics support, positively impacts cancer treatment service access [64–66]. As the Indigenous people often travels long to ET centres that are usually located in urban areas, the pre-arrangement of sustainable and adequately funded transport options, including reliable public or private transport systems, should be the priority of stakeholders. Financial expenses could also limit service access, and thus launching and/or strengthening a sustainable funding system to cover direct costs such as accommodation expenses and compensating indirect costs, including the loss of productivity, could be indispensable to improve ET utilisation [65, 66].

Ensuring culturally competent care, building trust in the health system, and providing tailored person-centred care [65, 67–69] are among the priority actions to sustainably transform Indigenous peoples’ health service utilisation, including ET use. Provision of culturally sensitive and trusted services could be achieved by working in collaboration with Indigenous community members [66], interventions on health care providers (e.g. Increasing the number of Indigenous health professionals and encouraging them to actively involve in the healthcare system, incorporating lessons about culturally competent patient care in the healthcare professionals training), construction and expansion of health services led by the Indigenous community [65–69]. A systematic review regarding interventions for the provision of culturally sensitive services reported the improvement of patients’ satisfaction upon culturally tailored programme interventions (e.g. education), improvement of service use among Indigenous patients when care is given by Indigenous health professionals, and increment of health professionals’ knowledge and confidence related to culturally competent service provision following training (e.g. education sessions, placement, group work) [67]. Further expansion of health care provision models targeted and/or led by the Indigenous people is also one of the strategies for growing culturally competent care and trustworthy health systems, thereby ultimately increasing uptake of cancer care, including ET use. For instance, in Australia, the Aboriginal Community-Controlled Health Services (ACCHSs) are established to address the Indigenous people’s health needs and provide a range of services such as culturally competent, accessible and comprehensive care, health promotion, culturally sensitive communication, employment and training, self-governance and problem-solving, and strengthening the national overall health system [68].

Furthermore, encouraging the participation of the Indigenous people in research and evidence-based practice by ensuring reliable data sources is one of the global Indigenous health agendas [4, 40], while this review highlighted the lack of diverse data sources for ET use (primary research focused data and secondary data). All articles used administrative databases (cancer registries and/or linkages with other administrative databases) that came from two developed countries: USA and New Zealand. To resolve the critical gaps regarding accurate, reliable and representative evidence based on a relatively large sample size concerning Indigenous peoples’ health [4, 40], it is important to work further on ensuring adequate inclusion of race/ethnicity, including Indigenous peoples, in widely used data sources (e.g. cancer registries). This is imperative because three quarters (74%) of the countries worldwide have cancer registries, with the majority having national coverage (42%) [70]. In addition, the coverage of cancer registries has increased over the past 50 years, and regional hubs have recently opened across the Pacific Islands, the Caribbean, Asia, Latin America, and Africa [71]. While the expansion of cancer registries is encouraging, it is also important to strengthen the efforts of maintaining cancer registries and establishing favourable data release and linkage policies; for example, nearly three-quarters of the articles in this review linked cancer registries with other administrative data sources to identify the Indigenous status and/or ET [72–74]. Not accessing articles published in countries with well-established cancer registries (e.g. Australia) could also be attributed to not registering race/ethnicity or ET in cancer registry or other relevant databases or policy barriers regarding data linkage and release to access data and conducting statistical analysis [72–74]. Administrative databases provide population-based data to monitor cancer care, including ET use, while they are not primarily established for research purposes and lack a comprehensive set of socioecological factors to understand the context or drivers of ET use [15, 29, 47, 48], such as lack of relevant interpersonal factors (such as social support, interaction with health professionals, cultural similarity) or community/organisational factors (such as health workers’ expertise, administrative guidelines and protocols, and patient support service availability) [15, 17–29], were noted in this review. Therefore, expanding primarily research-focused databases, including longitudinal data with a comprehensive set of factors, are important not only to understand the ET use and its related factors but also deemed to have implication in ensuring optimal cancer care and health equity among Indigenous peoples. Optimal utilisation and adherence of treatments in chronic disease including cancer is also one of the challenges among Indigenous peoples [16] and further research specifically focused on ET adherence and compliance is required.

This systematic review primarily aimed to collate the findings of articles on ET utilisation for cancer among Indigenous people and describe relevant factors that may influence ET use. It is also equally crucial to understand the level of adherence to endocrine treatment among Indigenous peoples with cancer diagnosis. For instance, two articles identified during our article screening process highlighted low adherence to endocrine treatment among Indigenous peoples compared to non-Indigenous peoples [31, 75], with statistically significant differences observed in one study [75]. The findings of these studies point towards the need for further comprehensive research that could provide adequate evidence on the level of ET adherence among Indigenous peoples with cancer diagnosis. Additionally, it is imperative to discuss about measurement issues when studying medication adherence. Additionally, it is imperative to discuss about measurement issues when studying medication adherence. For instance, the two studies mentioned above measured adherence similarly, defining it using an 80% medication possession ratio, which is the proportion of days covered by medication (calculated by dividing the number of days covered by prescriptions by the total number of days in the follow-up period) [31, 75].

Strengths and limitations.

To our knowledge, this review and meta-analysis is the first to comprehensively review and analyse existing evidence regarding the global rate of ET use and the distribution of factors that could influence ET use by Indigenous status. As a result of our comprehensive search methodology and inclusion criteria, we were able to include many articles conducted on Indigenous peoples. We also did all the possible sub-group analyses to investigate sources of heterogeneity. The majority of articles included exhibited good quality (fulfilled seven and above JBI’s critical appraisal) and were conducted using population-based data sources. However, this study has some limitations. Although we employed a rigorous search strategy to include all potential articles, it is still possible that we may have missed some studies that were not indexed in the included databases. Additionally, our decision to include only studies published in English may have introduced article selection bias, given that our study aimed to assess ‘worldwide’ outcomes. Furthermore, heterogeneity between studies was observed while using a random-effect model to account it. However, we have attempted to explore the source of heterogeneity through meta-regression, sensitivity, and sub-group analysis. It would also be important to reiterate the existing methodological limitation in estimating heterogeneity while conducting meta-analysis of proportions, which has been reported to usually show high heterogeneity [38, 39].

## Conclusion

Despite the higher rate of advanced cancer at diagnosis, Indigenous peoples exhibit lower ET utilisation than non-Indigenous populations. Compared to non-Indigenous populations, high proportion of Indigenous peoples live in rural areas and far from health facilities and have low socioeconomic status and high comorbidity rates. The reasons behind these disparities are not fully understood; however, occur alongside unequal access to cancer diagnosis and treatment services. Multifaceted interventions at individual, interpersonal, and community levels, including the expansion of cancer speciality services to rural and regional areas, provision of culturally appropriate and personalised care, and scaling up health service access, could be crucial to enhance the utilisation of ET and ultimately achieve the best possible cancer outcomes, as well as address unwarranted disparities. The limited data about the relationship between socioecological factors and ET use that was identified by this review highlights the need for further research to comprehensively examine personal and interpersonal factors that may influence ET use among Indigenous peoples with cancer.

### Electronic supplementary material

Below is the link to the electronic supplementary material.

Supplementary Material 1. Additional file 1. Completed PRISMA checklist.

Supplementary Material 2. Additional file 2. Searching terms used in the PubMed and CINAHL databases.

Supplementary Material 3. Additional file 3. Treatment and/or drug names used in the included articles for the systematic review.

Supplementary Material 4. Additional file 4. Includes a figure (Figure S1) that shows the pooled proportion of ET among ‘Indigenous/others’ (e.g., Asian/Pacific islanders), along with sensitivity analysis (Table S1), and all twenty-two studies reporting ET use (i.e., ‘Indigenous alone and Asian/Pacific Islanders’ (Figure S2)).

Supplementary Material 5. Additional file 5. Includes figure (A) that shows meta-analysis estimates, given named study is omitted; table (A) that shows a sensitivity analysis among Indigenous peoples, and table (B) that shows a sensitivity analysis among non-Indigenous populations (Note: only referring articles that separately reported the estimate in Indigenous peoples).

Supplementary Material 6. Additional file 6. Funnel plot examining publication bias for 13 Articles that reported ET use in Indigenous peoples.

Supplementary Material 7. Additional file 7. The critical appraisal results for articles included in the systematic review and meta-analysis.

## Data Availability

Published articles were the data sources for this study. The relevant data from articles are available within the manuscript and/or the online supplementary materials.

## Abbreviations

ADT: Androgen Deprivation Therapy
APIs: Asian Pacific Islanders
CI: Confidence Intervals
ET: Endocrine Treatment
LHRH: Luteinising Hormone-Releasing Hormone
HER2: Human Epidermal Growth Factor receptor 2
PRISMA: Preferred Reporting Items for Systematic Reviews and Meta-Analysis
