# Supplementary material for: Utilisation of endocrine therapy for cancer in Indigenous peoples: a systematic review and meta-analysis
Source: BMC Cancer. 2024 Jul 22;24:882. doi: 10.1186/s12885-024-12627-6 (PMC11264465; doi:10.1186/s12885-024-12627-6)
Supplement: Supplementary file 2 — Supplementary Material 2. Additional file 2. Searching terms used in the PubMed and CINAHL databases. [file 12885_2024_12627_MOESM2_ESM.docx]

**Utilisation of endocrine therapy for cancer in Indigenous peoples worldwide: a systematic review and meta-analysis**

Habtamu Mellie Bizuayehu^1#^, Sewunet Admasu Belachew^1#*^, Shafkat Jahan^1^, Abbey Diaz^1,4^, Siddharta Baxi^2^, Kalinda Griffiths^3,4,5^, Gail Garvey^1^

^1^ First Nations Cancer and Wellbeing (FNCW) Research Program, School of Public Health, The University of Queensland, Australia

^2^ GenesisCare Australia, Griffith University, Australia

^3^Poche SA+NT, Flinders University, Darwin, Australia

^4^Menzies School of Health Research, Darwin, Australia

^5^Centre for Big Data Research in Health, UNSW, Australia

^#^ Habtamu Mellie Bizuayehu and Sewunet Admasu Belachew are joint first authors as they contributed equally to this work.

**Corresponding author details:**

***Sewunet Admasu Belachew**

First Nations Cancer and Wellbeing (FNCW) Research Program, School of Public Health, The University of Queensland

**Email:** [s.admasubelachew@uq.edu.au](mailto:s.admasubelachew@uq.edu.au) |and| h.bizuayehu@uq.edu.au

**Twitter handle:** @BelacAdmasu; @HabtamuMellie

**Additional file 2: Searching terms used in PubMed and CINAHL databases for the systematic review.**

| **Database** | **Order** | **Search terms** | **Accessed articles** |
| --- | --- | --- | --- |
| **CINAHL** | S1 | TX “first nations” | 3,660 |
|  | S2 | MW alaska native | 103 |
|  | S3 | TX “alaska native*” | 8,609 |
|  | S4 | TX “American India” | 9 |
|  | S5 | TX "American nativecontinental ancestry" | 5 |
|  | S6 | TX "health services,indigenous" | 2,769 |
|  | S7 | TX "Indians, northAmerican" | 16 |
|  | S8 | TX aboriginal* | 17,880 |
|  | S9 | TX "native american" | 13,099 |
|  | S10 | TX "native indian" | 118 |
|  | S11 | TX "native canadian*" | 223 |
|  | S12 | MW inuit | 364 |
|  | S13 | TX inuit | 2,309 |
|  | S14 | TX metis | 1,022 |
|  | S15 | TX "pacific islander*" | 14,049 |
|  | S16 | TX samoa* | 4,030 |
|  | S17 | MW "american samoa" | 76 |
|  | S18 | TX "american samoa" | 1,245 |
|  | S19 | MW "pacific islands" | 1,375 |
|  | S20 | TX "pacific islands" | 2,667 |
|  | S21 | MW polynesia | 284 |
|  | S22 | TX polynesia | 997 |
|  | S23 | TX maori* | 9,684 |
|  | S24 | TX "Native Hawaiian*" | 4,416 |
|  | S25 | TX “Kanaka Maoli*” | 19 |
|  | S26 | TX aborigine* | 824 |
|  | S27 | TX "Torres StraitIslander*" | 4,938 |
|  | S28 | TX haida | 150 |
|  | S29 | TX cree | 4,037 |
|  | S30 | TX ojibwe | 132 |
|  | S31 | TX anishnawbe | 20 |
|  | S32 | TX anishinaabe* | 147 |
|  | S33 | TX mohawk* | 617 |
|  | S34 | TX dene | 1,911 |
|  | S35 | TX algonquin* | 396 |
|  | S36 | TX mississauga* | 10,157 |
|  | S37 | TX Seneca | 1,506 |
|  | S38 | S1 OR S2 OR S3 OR S4OR S5 OR S6 OR S7 ORS8 OR S9 OR S10 ORS11 OR S12 OR S13 ORS14 OR S15 OR S16 ORS17 OR S18 OR S19 ORS20 OR S21 OR S22 ORS23 OR S24 OR S25 ORS26 OR S27 OR S28 ORS29 OR S30 OR S31 ORS32 OR S33 OR S34 ORS35 OR S36 OR S37 | 81,583 |
|  | S39 | TX "endocrine therapy" | 5,297 |
|  | S40 | TX "endocrine treatment" | 1,138 |
|  | S41 | TX "hormon* therapy" | 19,831 |
|  | S42 | TX "hormon* treatment" | 4,071 |
|  | S43 | MW Aromatase Inhibitors | 2,246 |
|  | S44 | TX "Aromatase Inhibitor*" | 5,821 |
|  | S45 | MW anastrozole | 242 |
|  | S46 | TX anastrozol* | 2,049 |
|  | S47 | MW letrozole | 103 |
|  | S48 | TX letrozole | 2,627 |
|  | S49 | MW tamoxifen | 4,158 |
|  | S50 | TX tamoxifen* | 12,337 |
|  | S51 | MW fulvestrant | 84 |
|  | S52 | TX fulvestrant | 1,235 |
|  | S53 | MW goserelin | 306 |
|  | S54 | TX goserelin* | 1,067 |
|  | S55 | TX buserelin* | 163 |
|  | S56 | MW leuprolide | 464 |
|  | S57 | TX leuprolide | 1,443 |
|  | S58 | MW medroxyprogesterone | 1,551 |
|  | S59 | TX medroxyprogesterone* | 3,546 |
|  | S60 | TX "megestrol acetate" | 747 |
|  | S61 | S39 OR S40 OR S41 ORS42 OR S43 OR S44 ORS45 OR S46 OR S47 ORS48 OR S49 OR S50 ORS51 OR S52 OR S53 ORS54 OR S55 OR S56 ORS57 OR S58 OR S59 ORS60 | 41,705 |
|  | S62 | MW neoplasms | 532,776 |
|  | S63 | TX cancer* | 909,155 |
|  | S64 | S62 OR S63 | 1,050,133 |
|  | **S65** | **S38 AND S61 AND S64** | **827** |
| **PubMed** | NA | ((((((((((((((((((((((((((((((((((((((first nations [All Fields])) OR ("american indian or alaska native"[MeSH Terms])) OR ("american indian or alaska native"[All Fields])) OR ("american native continental ancestry group"[All Fields])) OR ("oceanic ancestry group"[All Fields])) OR ("indigenous"[All Fields])) OR ("health services, indigenous"[MeSH Terms])) OR ("indians, north american"[MeSH Terms])) OR ("north american indians"[All Fields])) OR ("aboriginal*"[All Fields])) OR (native 3 NEAR (indian* or american* or canadian*)) OR ("inuit"[MeSH Terms])) OR ("inuit*"[All Fields])) OR ("metis"[All Fields])) OR ("pacific islander*"[All Fields])) OR ("samoa*"[All Fields])) OR ("american samoa"[MeSH Terms])) OR ("american samoa"[All Fields])) OR ("pacific islands"[MeSH Terms])) OR ("pacific islands"[All Fields])) OR ("polynesia"[MeSH Terms])) OR ("polynesia"[All Fields])) OR ("maori*"[All Fields])) OR ("Native Hawaiian*"[All Fields])) OR (Kanaka Maoli*[All Fields])) OR ("aborigine*"[All Fields])) OR ("Torres Strait Islander*"[All Fields])) OR ("haida"[All Fields])) OR ("cree"[All Fields])) OR ("ojibwe"[All Fields])) OR ("anishnawbe"[All Fields])) OR ("anishinaabe*"[All Fields])) OR ("mohawk*"[All Fields])) OR ("dene"[All Fields])) OR ("algonquin*"[All Fields])) OR ("mississauga*"[All Fields])) OR ("seneca"[All Fields])) AND ((((((((((((((((((((((((((("hormones"[MeSH Terms]) AND ("therapeutics"[MeSH Terms])) OR ("endocrine therapy" [All Fields])) OR ("endocrine treatment"[All Fields])) OR ("hormone therapy"[All Fields])) OR ("hormone treatment"[All Fields])) OR ("Aromatase Inhibitors"[MeSH Terms])) OR ("Aromatase Inhibitor*"[All Fields])) OR ("anastrozole"[MeSH Terms])) OR ("anastrozol*"[All Fields])) OR ("letrozole"[MeSH Terms])) OR ("letrozole"[All Fields])) OR ("tamoxifen"[MeSH Terms])) OR ("tamoxifen*"[All Fields])) OR ("fulvestrant"[MeSH Terms])) OR ("fulvestrant"[All Fields])) OR ("goserelin"[MeSH Terms])) OR ("goserelin*"[All Fields])) OR ("buserelin"[MeSH Terms])) OR ("buserelin*"[All Fields])) OR ("leuprolide"[MeSH Terms])) OR ("leuprolide"[All Fields])) OR ("leuprorelin"[All Fields])) OR ("medroxyprogesterone"[MeSH Terms])) OR ("medroxyprogesterone*"[All Fields])) OR ("megestrol acetate"[MeSH Terms])) OR ("megestrol acetate"[All Fields])) AND ((neoplasms[MeSH Terms]) OR (cancer*[Title/Abstract]))) | 111 |

NA=not applicable
