# Supplementary material for: Utilisation of endocrine therapy for cancer in Indigenous peoples: a systematic review and meta-analysis
Source: BMC Cancer. 2024 Jul 22;24:882. doi: 10.1186/s12885-024-12627-6 (PMC11264465; doi:10.1186/s12885-024-12627-6)

**Utilisation of endocrine therapy for cancer in Indigenous peoples worldwide: a systematic review and meta-analysis**

Habtamu Mellie Bizuayehu^1#^, Sewunet Admasu Belachew^1#*^, Shafkat Jahan^1^, Abbey Diaz^1,4^, Siddharta Baxi^2^, Kalinda Griffiths^3,4,5^, Gail Garvey^1^

^1^ First Nations Cancer and Wellbeing (FNCW) Research Program, School of Public Health, The University of Queensland, Australia

^2^ GenesisCare Australia, Griffith University, Australia

^3^Poche SA+NT, Flinders University, Darwin, Australia

^4^Menzies School of Health Research, Darwin, Australia

^5^Centre for Big Data Research in Health, UNSW, Australia

^#^Habtamu Mellie Bizuayehu and Sewunet Admasu Belachew are joint first authors as they contributed equally to this work.

**Corresponding author details:**

***Sewunet Admasu Belachew**

First Nations Cancer and Wellbeing (FNCW) Research Program, School of Public Health, The University of Queensland

**Email:** [s.admasubelachew@uq.edu.au](mailto:s.admasubelachew@uq.edu.au) |and| h.bizuayehu@uq.edu.au

**Twitter handle:** @BelacAdmasu; @HabtamuMellie

**Additional file 3: Treatment and/or drug names used in the included articles for the systematic review.**


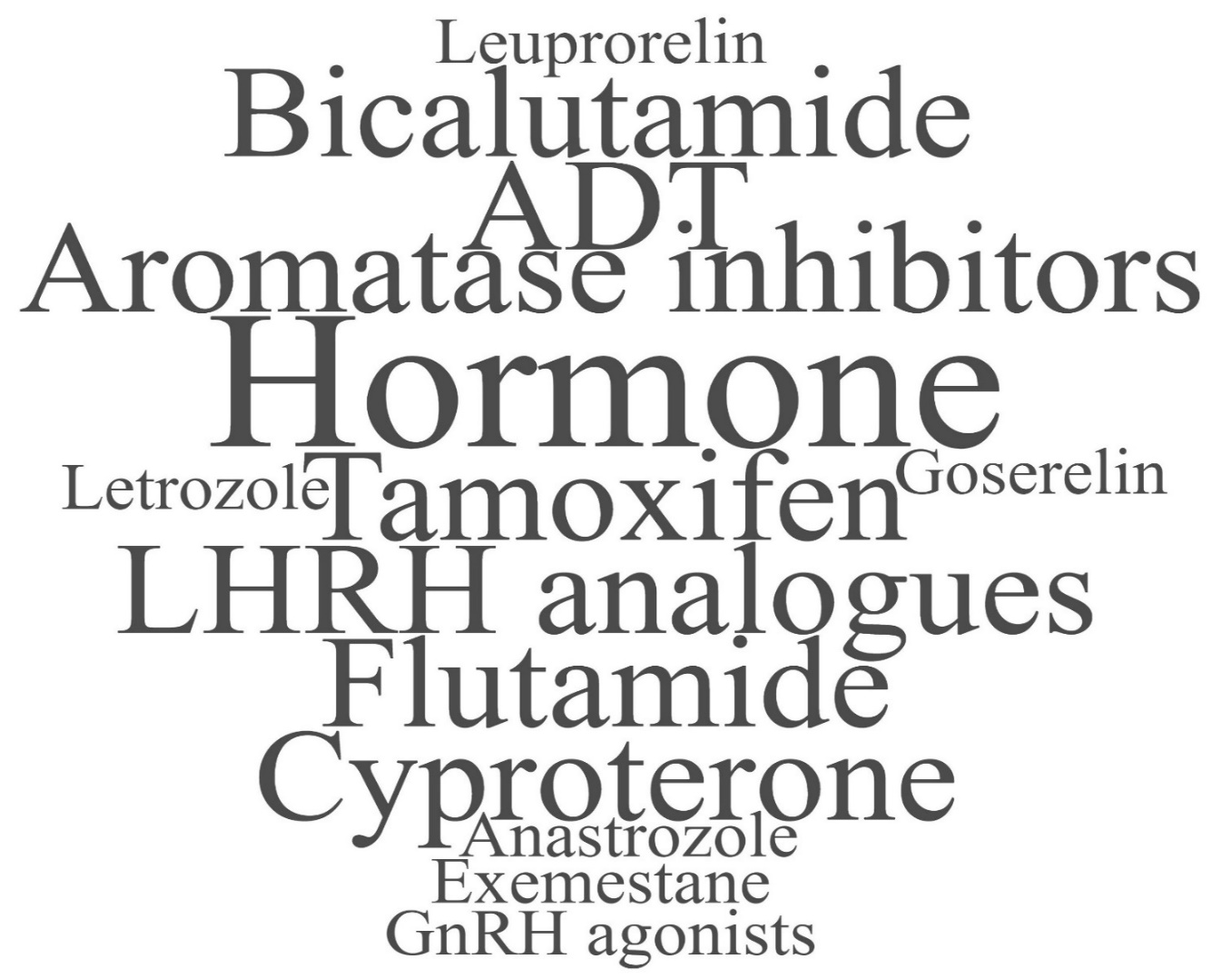

Supplement: Supplementary file 3 — Supplementary Material 3. Additional file 3. Treatment and/or drug names used in the included articles for the systematic review. [file 12885_2024_12627_MOESM3_ESM.docx]
