# Supplementary material for: Utilisation of endocrine therapy for cancer in Indigenous peoples: a systematic review and meta-analysis
Source: BMC Cancer. 2024 Jul 22;24:882. doi: 10.1186/s12885-024-12627-6 (PMC11264465; doi:10.1186/s12885-024-12627-6)

**Utilisation of endocrine therapy for cancer in Indigenous peoples worldwide: a systematic review and meta-analysis**

Habtamu Mellie Bizuayehu^1#^, Sewunet Admasu Belachew^1#*^, Shafkat Jahan^1^, Abbey Diaz^1,4^, Siddharta Baxi^2^, Kalinda Griffiths^3,4,5^, Gail Garvey^1^

^1^ First Nations Cancer and Wellbeing (FNCW) Research Program, School of Public Health, The University of Queensland, Australia

^2^ GenesisCare Australia, Griffith University, Australia

^3^Poche SA+NT, Flinders University, Darwin, Australia

^4^Menzies School of Health Research, Darwin, Australia

^5^Centre for Big Data Research in Health, UNSW, Australia

^#^Habtamu Mellie Bizuayehu and Sewunet Admasu Belachew are joint first authors as they contributed equally to this work.

**Corresponding author details:**

***Sewunet Admasu Belachew**

First Nations Cancer and Wellbeing (FNCW) Research Program, School of Public Health, The University of Queensland

**Email:** [s.admasubelachew@uq.edu.au](mailto:s.admasubelachew@uq.edu.au) |and| h.bizuayehu@uq.edu.au

**Twitter handle:** @BelacAdmasu; @HabtamuMellie

**Additional file 4: A figure (Figure S1) that shows the pooled proportion of ET use among articles that reported peoples diagnosed with cancer as ‘Indigenous/others’ (e.g. Asian/Pacific islanders), the table (Table S1) below shows the estimated proportion when articles with outlier values are excluded, and in all twenty-two studies reporting ET use (i.e. ‘Indigenous alone and Asian/Pacific Islanders’ (Figure S2 ).**

**Figure S1**


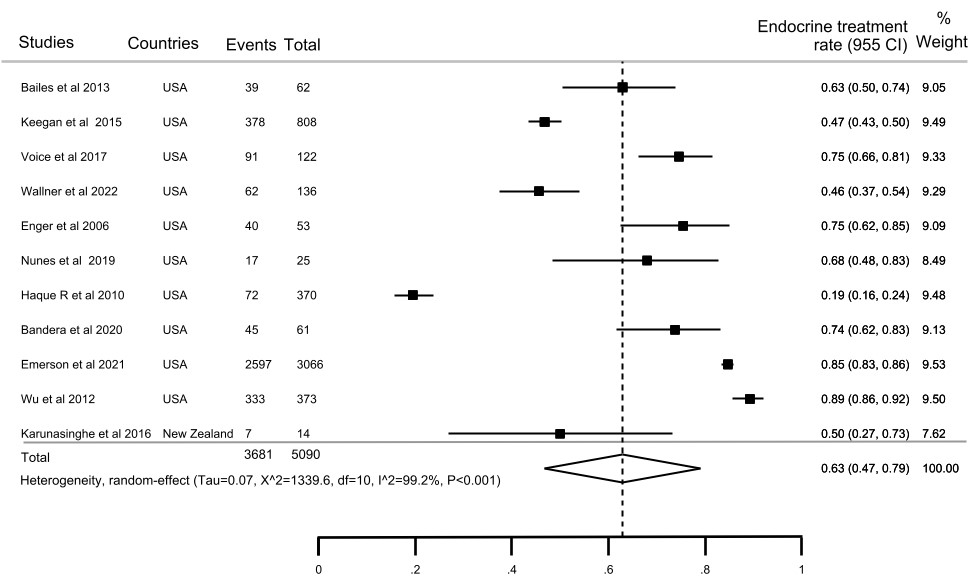


**Table S1**

| **Number of articles with outlier values omitted** | **Articles for the estimate** | **Endocrine utilisation rate, %** | **95% confidence interval** | **Heterogeneity** |
| --- | --- | --- | --- | --- |
| Three [*Haque et al 2010, Wallner et al 2022, Keegan et al 2015*] | 8 | 77 | 71-83 | I^2^ =83.8%, *P*<0.001 |

**Figure S2**


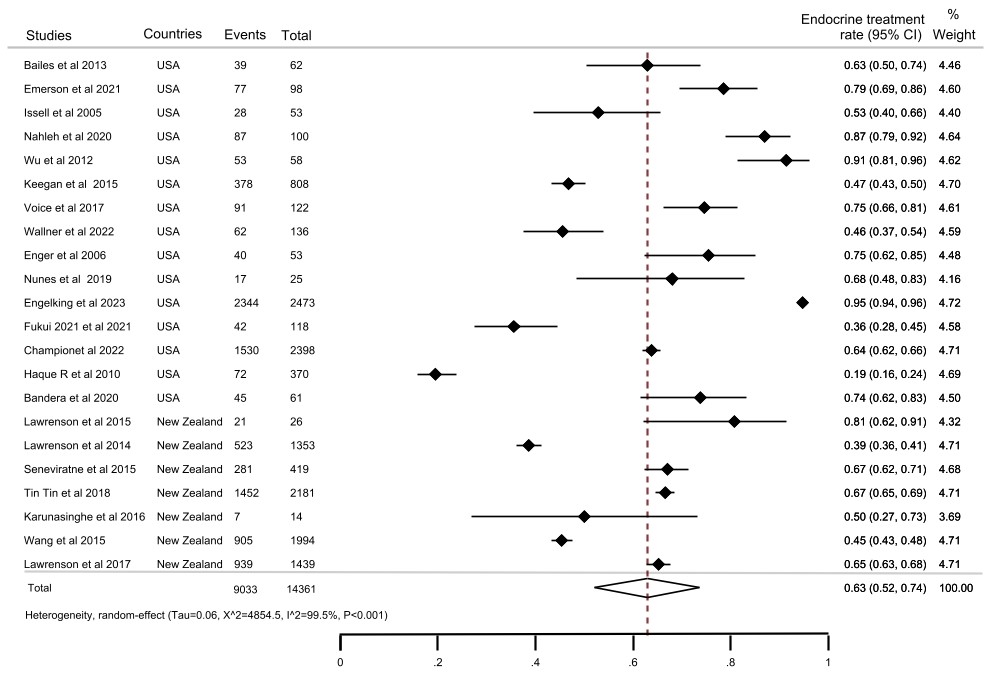

Supplement: Supplementary file 4 — Supplementary Material 4. Additional file 4. Includes a figure (Figure S1) that shows the pooled proportion of ET among ‘Indigenous/others’ (e.g., Asian/Pacific islanders), along with sensitivity analysis (Table S1), and all twenty-two studies reporting ET use (i.e., ‘Indigenous alone and Asian/Pacific Islanders’ (Figure S2)). [file 12885_2024_12627_MOESM4_ESM.docx]
