# Supplementary material for: Utilisation of endocrine therapy for cancer in Indigenous peoples: a systematic review and meta-analysis
Source: BMC Cancer. 2024 Jul 22;24:882. doi: 10.1186/s12885-024-12627-6 (PMC11264465; doi:10.1186/s12885-024-12627-6)
Supplement: Supplementary file 5 — Supplementary Material 5. Additional file 5. Includes figure (A) that shows meta-analysis estimates, given named study is omitted; table (A) that shows a sensitivity analysis among Indigenous peoples, and table (B) that shows a sensitivity analysis among non-Indigenous populations (Note: only referring articles that separately reported the estimate in Indigenous peoples). [file 12885_2024_12627_MOESM5_ESM.docx]

**Utilisation of endocrine therapy for cancer in Indigenous peoples worldwide: a systematic review and meta-analysis**

Habtamu Mellie Bizuayehu^1#^, Sewunet Admasu Belachew^1#*^, Shafkat Jahan^1^, Abbey Diaz^1,4^, Siddharta Baxi^2^, Kalinda Griffiths^3,4,5^, Gail Garvey^1^

^1^ First Nations Cancer and Wellbeing (FNCW) Research Program, School of Public Health, The University of Queensland

^2^ GenesisCare Australia, Griffith University, Australia

^3^Poche SA+NT, Flinders University, Darwin, Australia

^4^Menzies School of Health Research, Darwin, Australia

^5^Centre for Big Data Research in Health, UNSW, Australia

^#^Habtamu Mellie Bizuayehu and Sewunet Admasu Belachew are joint first authors as they contributed equally to this work.

**Corresponding author details:**

***Sewunet Admasu Belachew**

First Nations Cancer and Wellbeing (FNCW) Research Program, School of Public Health, The University of Queensland

**Email:** [s.admasubelachew@uq.edu.au](mailto:s.admasubelachew@uq.edu.au) |and| h.bizuayehu@uq.edu.au

**Twitter handle:** @BelacAdmasu; @HabtamuMellie

**Additional file 5**: Includes figure (A) that shows meta-analysis estimates, given named study is omitted; table (A) that shows a sensitivity analysis among Indigenous peoples, and table (B) that shows a sensitivity analysis among non-Indigenous populations (Note: only referring articles that separately reported the estimate in Indigenous peoples)

**Figure (A)**


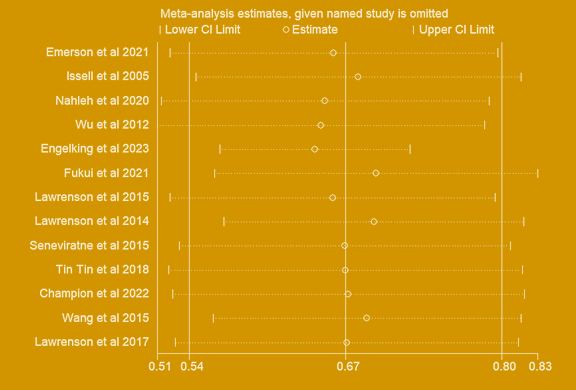


**Table (A)**

| **Number of articles with outlier values omitted** | **Articles for the estimate** | **Endocrine Rx utilisation rate, %** | **95% confidence interval** | **Heterogeneity** |
| --- | --- | --- | --- | --- |
| Three [*Engelking et al 2023, Fukui et al 2021, Lawrenson et al 2014*] | Ten | 70 | 62-77 | I^2^ =97.7%, *P*<0.001 |
| Five [*Engelking et al 2023, Fukui et al 2021, Lawrenson et al 2014, Nahleh et al 2020, Wu et al 2012, and Wang et al 2015*] | Eight | 67 | 64-69 | I^2^ =72.5%, *P*<0.001 |

**Table (B)**

| **Number of articles with outlier values omitted** | **Articles for the estimate** | **Endocrine utilisation rate, %** | **95% confidence interval** | **Heterogeneity** |
| --- | --- | --- | --- | --- |
| Four [*Engelking et al 2023, Fukui et al 2021*, *Wu et al 2012, Wang et al 2015*] | 4 | 72 | 58-86 | I^2^ =99.9%, *P*<0.001 |
