# Supplementary material for: Utilisation of endocrine therapy for cancer in Indigenous peoples: a systematic review and meta-analysis
Source: BMC Cancer. 2024 Jul 22;24:882. doi: 10.1186/s12885-024-12627-6 (PMC11264465; doi:10.1186/s12885-024-12627-6)
Supplement: Supplementary file 7 — Supplementary Material 7. Additional file 7. The critical appraisal results for articles included in the systematic review and meta-analysis. [file 12885_2024_12627_MOESM7_ESM.docx]

**Utilisation of endocrine therapy for cancer in Indigenous peoples worldwide: a systematic review and meta-analysis**

Habtamu Mellie Bizuayehu^1#^, Sewunet Admasu Belachew^1#*^, Shafkat Jahan^1^, Abbey Diaz^1,4^, Siddharta Baxi^2^, Kalinda Griffiths^3,4,5^, Gail Garvey^1^

^1^ First Nations Cancer and Wellbeing (FNCW) Research Program, School of Public Health, The University of Queensland

^2^ GenesisCare Australia, Griffith University, Australia

^3^Poche SA+NT, Flinders University, Darwin, Australia

^4^Menzies School of Health Research, Darwin, Australia

^5^Centre for Big Data Research in Health, UNSW, Australia

^#^ Habtamu Mellie Bizuayehu and Sewunet Admasu Belachew are joint first authors as they contributed equally to this work.

**Corresponding author details:**

***Sewunet Admasu Belachew**

First Nations Cancer and Wellbeing (FNCW) Research Program, School of Public Health, The University of Queensland

**Email:** [s.admasubelachew@uq.edu.au](mailto:s.admasubelachew@uq.edu.au) |and| h.bizuayehu@uq.edu.au

**Twitter handle:** @BelacAdmasu; @HabtamuMellie

**Additional file 6: The critical appraisal results for articles included in the systematic review and meta-analysis**

| **No** | **Criteria** | **First author name and year of publication** | | | | | | | | | | | | | | | | | | | | | | | | | | | | | |
| --- | --- | --- | --- | --- | --- | --- | --- | --- | --- | --- | --- | --- | --- | --- | --- | --- | --- | --- | --- | --- | --- | --- | --- | --- | --- | --- | --- | --- | --- | --- | --- |
|  |  | **Bailes, 2013 [30]** | **Blackmore, 2018 [79]** | **Emerson, 2021** **[31]** | **Engelking, 2023** **[58]** | **Fukui, 2021 [76]** | **Lawrenson, 2014 [32]** | **Lawrenson, 2015 [32]** | **Lawrenson, 2017 [80]** | **Seneviratne, 2015 [24]** | **Tin Tin, 2018 [60]** | **Champion, 2022 [57]** | **Wang, 2015 [82]** | **Bandera, 2020 [59]** | **Enger, 2006 [33]** | **Karunasinghe, 2016 [34]** | **Keegan, 2015 [55]** | **Nunes, 2019 [78]** | **Voci, 2018 [25]** | **Wallner, 2022 [75]** | **Issell, 2005 [35]** | **Haque, 2010 [84]** | | **Nahleh, 2020 [77]** | | **Wu, 2012 [26]** | | **Prehn, 2002 [36]** | |  |  |
| 1 | Was the sample frame appropriate to address the target population? | Y | Y | Y | Y | Y | Y | Y | Y | Y | Y | Y | Y | Y | Y | Y | Y | Y | Y | Y | Y | | Y | | Y | | Y | | Y | |  |
| 2 | Were study participants sampled in an appropriate way? | Y | Y | Y | Y | Y | Y | Y | Y | Y | Y | Y | Y | Y | Y | U | Y | Y | Y | Y | Y | | Y | | Y | | Y | | Y | |  |
| 3 | Was the sample size adequate? | Y | Y | Y | Y | N | Y | N | Y | Y | Y | Y | Y | Y | Y | N | Y | Y | Y | Y | Y | | Y | | Y | | Y | | Y | |  |
| 4 | Were the study subjects and the setting described in detail? | Y | Y | Y | Y | Y | Y | Y | Y | Y | Y | Y | Y | Y | Y | Y | Y | Y | Y | Y | Y | | Y | | Y | | Y | | Y | |  |
| 5 | Was the data analysis conducted with sufficient coverage of the identified sample? | Y | Y | Y | Y | Y | Y | Y | Y | Y | Y | Y | Y | Y | Y | Y | Y | Y | Y | Y | Y | | Y | | Y | | Y | | Y | |  |
| 6 | Were valid methods used for the identification of the condition? | Y | Y | Y | Y | Y | Y | Y | Y | Y | N | Y | Y | Y | Y | Y | Y | Y | Y | Y | Y | | Y | | Y | | Y | | Y | |  |
| 7 | Was the condition measured in a standard, reliable way for all participants? | Y | Y | Y | Y | Y | Y | Y | Y | Y | Y | Y | Y | Y | Y | Y | Y | Y | Y | Y | Y | | Y | | Y | | Y | | Y | |  |
| 8 | Was there appropriate statistical analysis? | Y | N | Y | Y | N | N | Y | N | Y | Y | N | N | U | N | N | N | N | Y | Y | Y | | N | | Y | | Y | | U | |  |
| 9 | Was the response rate adequate, and if not, was the low response rate managed appropriately? | Y | Y | Y | Y | Y | Y | Y | Y | Y | Y | Y | Y | Y | Y | Y | Y | Y | Y | Y | Y | | Y | | Y | | Y | | Y | |  |
|  | **Total score (max 9)** | 9 | 8 | 9 | 9 | 7 | 8 | 8 | 8 | 9 | 8 | 8 | 8 | 8 | 8 | 7 | 8 | 8 | 9 | 9 | 9 | | 8 | | 9 | | 9 | | 8 | |  |

**Responses: N:** no **Y:** yes **NA:** Not applicable **U:** unclear
